# Weight Stigma Amongst Nurses and Nursing Students: A Scoping Review of Direct and Comparative Evidence

**DOI:** 10.1111/jan.16843

**Published:** 2025-02-24

**Authors:** Mahshid Fonoudi, Beverley Ewens, Amanda Towell‐Barnard

**Affiliations:** ^1^ School of Nursing and Midwifery Edith Cowan University Joondalup Western Australia Australia

**Keywords:** nurses, nursing care, scoping review, weight bias, weight stigma

## Abstract

**Aim:**

To map both direct and comparative research on weight stigma amongst nurses and nursing students by identifying the extent, range and nature of studies and identify the gaps in this area.

**Design:**

Scoping review, following the Joanna Briggs Institute (JBI) guidelines.

**Data Sources:**

Seven databases including MEDLINE, Web of Science, CINAHL, Embase, Scopus, PsycINFO and Cochrane Library, in addition to Google Scholar and Open Access Theses and Dissertations were systematically searched in August 2023.

**Methods:**

Inclusion criteria comprised nurses or nursing students as participants, weight stigma as the concept and any context. After uploading all search results into EndNote and removing duplicates, titles and abstracts/full‐texts were screened. One reviewer extracted data, which were checked and confirmed by other authors. Data were analysed using frequency counts, numerical range and inductive open coding and then reported through diagrams, tables and a narrative summary.

**Results:**

From 2213 initial search results, 80 studies were included. The range of studies regarding their characteristics was described. Studies were mapped in terms of objectives and findings and eight descriptors were identified; including description, comparing different groups of nurses, exploring associations, intervention assessment, comparing nurses with other health professionals, exploring consequences or causes, psychometrics and finding solutions.

**Conclusion:**

The majority of included studies were conducted in the United States, had a cross‐sectional design, and included a high percentage of female participants. Future research with more diversity in terms of participants' gender and country, qualitative designs and a focus on practical strategies to reduce weight stigma will improve the understanding of weight stigma in nursing care.

**Impact:**

The identified gaps in this study can guide future research to develop more practical strategies to reduce weight stigma amongst nurses, modify nursing education and provide relevant healthcare policies. Consequently, the quality of care for higher weight individuals may be improved.

**Reporting Method:**

The EQUATOR guidelines for PRISMA have been utilised.

**Patient or Public Contribution:**

None.

## 1 Introduction

Stigma is the social phenomenon of targeting groups (Andersen et al. 2022) which starts with the identification of perceived differences in people such as physical features (Link and Phelan 2006), behavioural patterns and group affiliations (Major and O'Brien 2005). Research has shown stigma towards multiple marginalised characteristics, including mental health issues (Pescosolido et al. 2021) and disability (Çaynak et al. 2022), amongst others. Higher weight individuals are another group of people who are frequently stigmatised. Almost 650 million adults worldwide were affected by obesity in 2016, statistics which had nearly tripled from 1975 (World‐Health‐Organization 2021). Weight stigma is a combination of negative stereotypical beliefs (such as laziness), prejudiced attitudes (such as feelings of disgust) and discriminatory behaviours (such as rejection in social settings) towards higher weight individuals (Papadopoulos et al. 2021). Weight stigma is also intertwined with the societal idealisation of thin bodies, linking obesity to character deficits (Klaczynski and Felmban 2019) and internalising thin ideals (Nutter et al. 2021).

The discourse in relation to the formulation of obesity is mainly biomedical (Bessey and Lordly 2020), focusing on the physiological consequences, such as cardiovascular disease, musculoskeletal disorders and some cancers (World‐Health‐Organization 2021). The psychosocial aspects of living in a larger body in the form of weight discrimination are associated with lower levels of educational success, housing (Puhl and Brownell 2001), employment (Bartels and Nordstrom 2013), income (Cawley 2004), psychological well‐being (Jackson et al. 2015) and shorter life expectancy (Sutin et al. 2015). However, these factors are mostly overlooked due to a culture of blame towards individuals for their higher weight (Puhl and Heuer 2010). Weight stigma has been identified in several groups of health care professionals as well, including dietitians (Diversi et al. 2016), paediatric nurses, clinical support staff (Garcia et al. 2016), primary care health professionals including nurses and doctors (Akman et al. 2010), physiotherapists (Setchell et al. 2014) and health care students (Swift et al. 2013). Weight controllability belief, or believing that weight is under one's control can intensify weight stigma (Tanneberger and Ciupitu‐Plath 2018).

## 2 Background

Health professionals are less likely to respect and educate higher weight individuals, and more likely to attribute health problems to their weight (Alberga et al. 2019), which may influence the quality of care delivered. In a study using hypothetical scenarios of patients with different body mass index (BMI) and similar conditions, health professionals of which 87% were nurses suggested weight loss plans, spent less time on teaching healthy lifestyle behaviours using positive terms, and used pharmaceutical treatments earlier for higher weight individuals compared to their lower weight counterparts (Seymour et al. 2018). Different treatment of patients based on their weight is considered unethical and a form of malpractice, as it can result in misdiagnosis (Chrisler and Barney 2017).

If higher weight individuals perceive poor treatment from health care professionals, they are more likely to avoid or delay using those facilities in the future (Alberga et al. 2019; Phelan et al. 2015a). Higher weight individuals report non‐verbal and verbal miscommunication, being told that weight loss will cure their symptoms, and encountering inadequacies in the physical environment for example, lack of appropriate furniture and medical equipment, in health care settings (O'Donoghue et al. 2021). These factors that may pose risks to the patients' health due to their size seem at odds with the purpose of medical treatment. Therefore, despite the good intentions of health professionals, they may unintentionally cause harm to higher weight individuals.

Nurses constitute the largest group of health professionals globally, at almost 59% of the health workforce (World‐Health‐Organization 2020); which indicates the importance of the care they deliver and the longer time they potentially spend with patients. In nursing care settings, caring for a patient with a larger body may be physically exhausting (Culbertson and Smolen 1999; Poon and Tarrant 2009), because of challenges such as moving heavier patients or the perceived risk of injuries to them (Shea and Gagnon 2015). It can also be emotionally frustrating for nurses (Dunagan et al. 2016), generating feelings of repulsion towards their patients with obesity (Puhl and Brownell 2001). One study demonstrated that more nurses than physicians lacked the desire to work with higher weight individuals or provide care to them. Additionally, nurses had negative perceptions about higher weight individuals, recounting that these patients were unable to carry out tasks on a daily basis or that they were not on time for medical appointments (Akman et al. 2010). Weight stigmatising beliefs may impact nurses' actions in practice and delivery of care, presenting a range of physical and emotional challenges for them. Despite the importance of nursing care for higher weight individuals, there is a paucity of evidence which focuses on weight stigma amongst nurses and nursing students. This review aimed to map both direct and comparative research on weight stigma amongst nurses and nursing students in terms of the extent, range and nature of existing studies, and to identify the gaps in this area, using a scoping review methodology. Throughout this article, the terms direct and comparative research will be used to refer to studies including only nursing populations and the ones including different health care groups, respectively.

## 3 The Review

### 3.1 Aim

This scoping review mapped both direct and comparative research on weight stigma amongst nurses and nursing students by identifying the extent, range and nature of studies and identified the gaps in this research area. The research question was ‘What is the extent, range, and nature of current research about weight stigma amongst nurses and nursing students?’

### 3.2 Methods

The Joanna Briggs Institute (JBI) manual for evidence synthesis was used to guide this review (Peters et al. 2020), and the Preferred Reporting Items for Systematic Reviews and Meta‐analyses Extension for Scoping Reviews (PRISMA‐ScR) was utilised to report it (Tricco et al. 2018). In September 2023, a protocol for this scoping review was registered on the Open Science Framework (OSF) website^1^.

### 3.3 Search Strategy

A preliminary search of MEDLINE was conducted first to identify appropriate keywords and index terms, including MeSH terms. They were then adapted and used for the second step which was a comprehensive search strategy for the following databases: MEDLINE, Web of Science, CINAHL, Embase, Scopus, PsycINFO and Cochrane Library. Search terms consisted of weight stigma, nurses, health care professionals and synonyms of these terms. In the third step, Google Scholar and Open Access Theses and Dissertations (OATD) were searched to locate theses. Finally, the reference lists of all included sources of evidence and excluded review articles were screened to find additional articles. Search alerts were activated in all databases in August 2023, and they were checked until June 2024. A sample of the search strategy used in MEDLINE is demonstrated in Appendix S1.

### 3.4 Eligibility Criteria

This scoping review was based upon the participant, concept and context (PCC) framework (Peters et al. 2020). Studies that included any group of nurses/nursing students as participants were considered. Studies where the population comprised different groups of health care professionals or students were included if the data could be extracted specifically from nurses or nursing students. This included both studies that focused directly on nursing populations as well as those with comparative designs that examined nurses/nursing students alongside other health care groups. Regarding concept, studies that considered any aspect of weight stigma (or closely related concepts, such as negative attitudes and behaviours towards higher weight individuals) were included in this review. No limitations on contextual characteristics were imposed, including location, ethnicity or gender (Appendix S2). The English language was selected for inclusion, without any constraints on time or study design. Only published peer‐reviewed research articles and theses were considered, whilst grey literature, conference abstracts, opinion papers and literature reviews were excluded.

### 3.5 Study Selection

**FIGURE 1:**
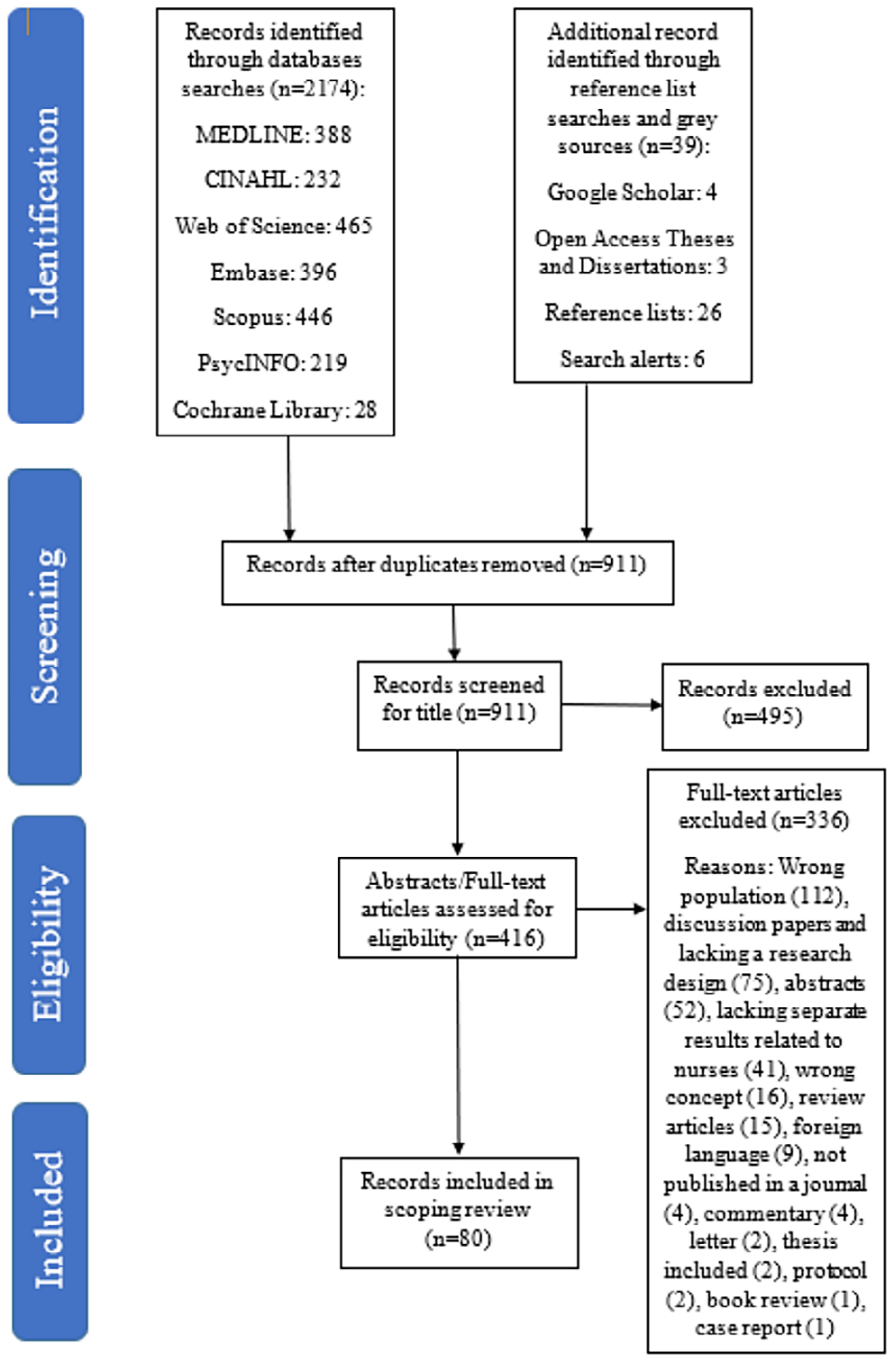
PRISMA flow diagram of search strategies (Tricco et al. 2018).

The number of retrieved articles and theses after the search was 2213, however, 2133 of them were excluded in the three stages of removing duplicates in EndNote 20 (Clarivate Analytics, USA), followed by title and then abstract/full‐text blind screening in Rayyan (Ouzzani et al. 2016). Each citation was screened by at least two independent reviewers in both screening stages (MF and BE or MF and ATB), and conflicts were resolved by asking the third reviewer's opinion, leaving 80 studies for inclusion. The PRISMA‐ScR (Tricco et al. 2018) is demonstrated using the PRISMA flow diagram (Figure 1).

### 3.6 Quality Appraisal

Quality appraisal is not a mandatory step in scoping reviews within the JBI guidelines, unless authors choose to assess and report the risk of bias in relation to their review objectives (Peters et al. 2020). Given the aim of this scoping review and our inclusion of only peer‐reviewed articles and theses, no formal quality appraisal was conducted.

### 3.7 Data Charting and Extraction

Data from sources of evidence were extracted according to the template for data extraction by JBI (Peters et al. 2020), which was modified according to the current research question. Location of care was added to the version of this table from our protocol, as it was reported in some included studies, and the focus of the study was removed and represented in a separate table as descriptors of the nature of the studies. Data extraction was conducted by one reviewer and confirmed by the other two authors. Key findings related to any group of nurses/nursing students and weight stigma were extracted and presented descriptively. When extracting data from comparative studies including different groups of health professionals, in some cases it was necessary to include contextual information from other health care groups. However, this proportion of data was limited to ‘study aims and objectives’ and ‘relevant key findings’; data were only extracted if they were closely intertwined with the nursing data in comparative settings. This approach ensured that a nursing‐specific focus was maintained throughout the study.

### 3.8 Data Analysis

Data were analysed using both quantitative and qualitative methods. Regarding the extent of research, frequency counts of included studies based on their publication types were reported. The range of studies was presented through a description of year of publication, countries, number of participants and their characteristics, areas of practice, study concepts, study designs and instruments used, using frequency counts or numerical range where applicable. Finally, the nature of studies was mapped by open coding of each objective and relevant key findings using an inductive approach, in terms of the focus of studies, followed by a categorisation of common concepts into themes (Pollock et al. 2023). A table showing how each evidence source added information to each category (Appendix S4), and a diagram of study categories indicating the nature of studies (Figure 4) were provided. Results were reported through diagrammatic and tabular presentations, accompanied by a narrative summary (Peters et al. 2020).

## 4 Results

### 4.1 The Extent and Range of the Research

As shown in Figure 1, the initial search in August 2023 resulted in more than two thousand articles and theses, with 80 being included after screening and checking for eligibility. The majority of sources were journal articles (*n* = 62), followed by doctoral dissertations (*n* = 11), master's theses (*n* = 4), honours' theses (*n* = 2) and bachelor's theses (*n* = 1). A summary of the study results is shown in Appendix S3.

**FIGURE 2:**
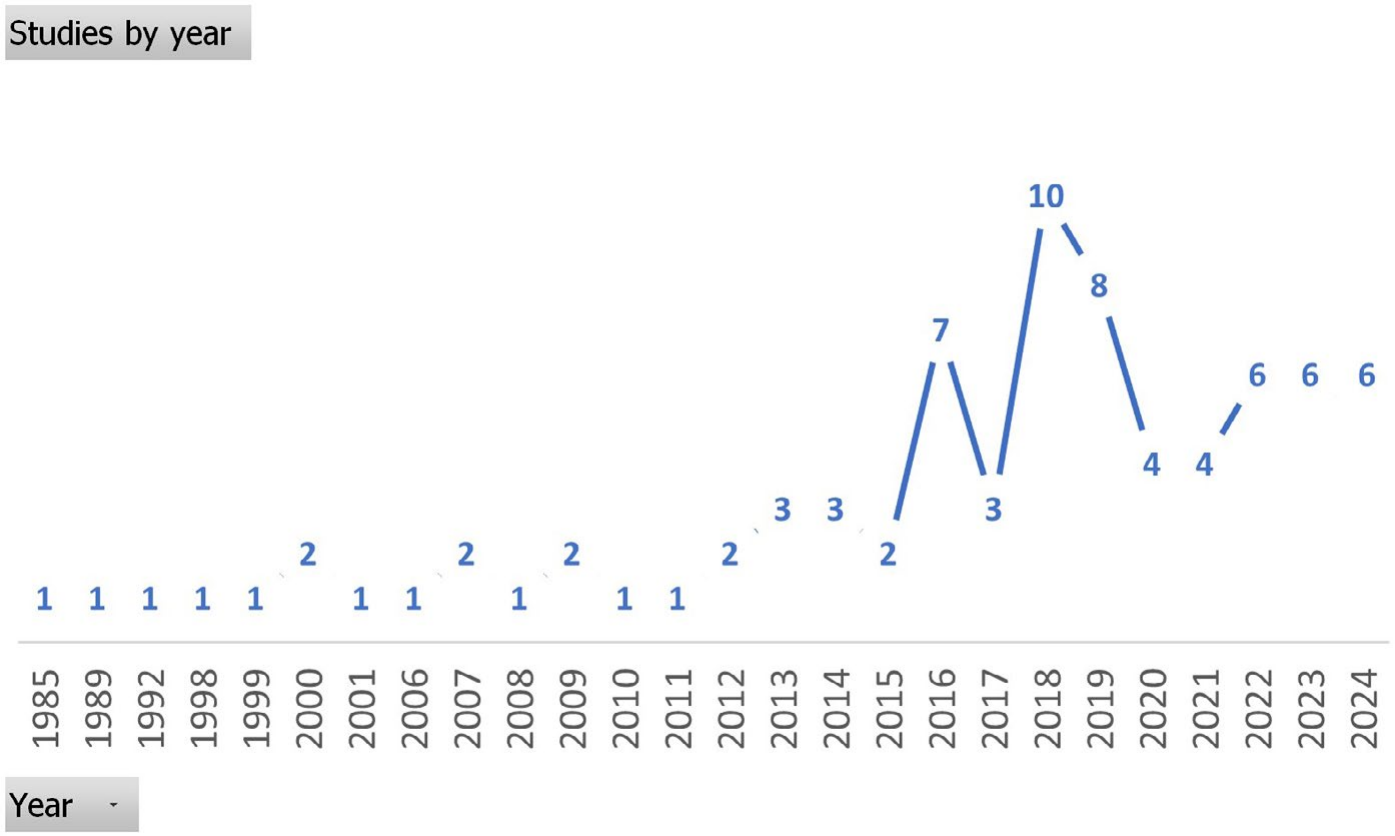
Studies published per year.

Publication years of studies ranged from 1985 to 2024. More than three quarters were published in the past decade, with 62 articles or theses published from 2013 to 2024. Figure 2 illustrates studies by year.

The number of studies rose markedly from 2016, reaching its peak in 2018, when ten studies were published. This graph also illustrates the significant proportion of published studies in the past decade.

Forty‐six studies were conducted in the US, with the other 34 conducted in Turkey (*n* = 7), the UK (*n* = 6), Germany (*n* = 3), Norway (*N* = 3), Canada (*n* = 3), Australia (*n* = 1), China (*n* = 1), Iran (*n* = 1), Malaysia (*n* = 1), the Netherlands (*n* = 1), Switzerland (*n* = 1), Namibia (*n* = 1), Hong Kong (*n* = 1), Spain (*n* = 1), Sweden (*n* = 1), Poland (*n* = 1) and Costa Rica (*n* = 1).

**FIGURE 3:**
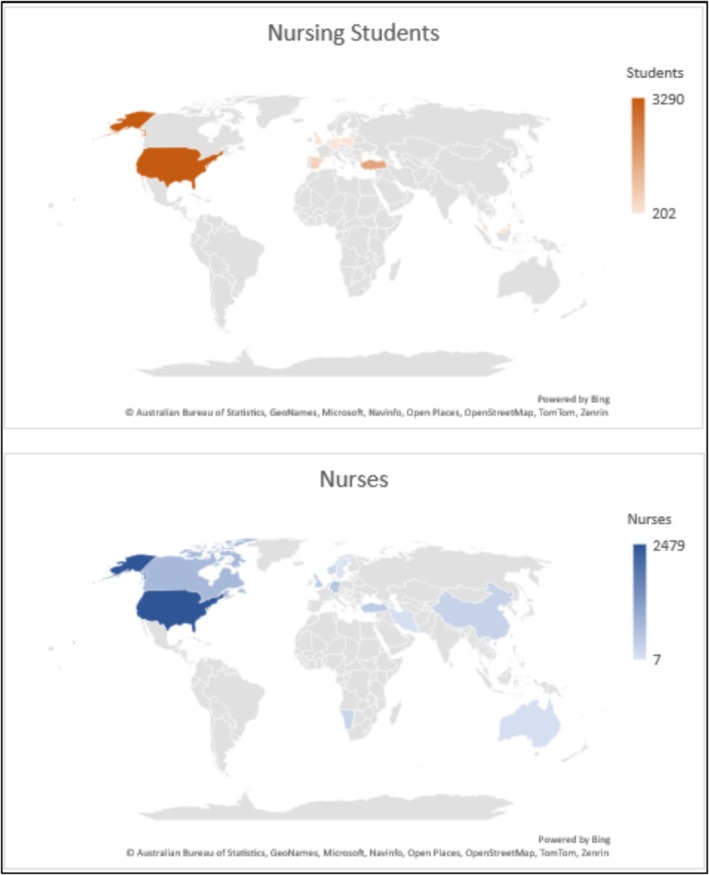
Geographical distribution of participants.

Participants included various groups of nurses (*n* = 38), nursing students (*n* = 37) or both (*n* = 5). Students comprised undergraduate students, master's students, nurse practitioner (NP) students and adult and mental health nursing students. Nurses were registered nurses (RNs), licence practical nurses (LPNs), certified nursing assistants (CNAs), NPs, intensive care unit (ICU) nurses, paediatric surgery nurses, school nurses, rehabilitation nurses, youth healthcare nurses, primary care nurses, nursing instructors, psychiatric RNs, registered nurse anaesthetists (RNAs) and clinical nurse specialists (CNSs). Amongst studies that provided information about gender ratio, most had a considerably higher percentage of female participants compared to male or non‐binary counterparts. The number of participants ranged from 7 to 685 nurses/nursing students. Figure 3 depicts the total number of participants from each country, including both nurses and nursing students.

As Figure 3 demonstrates, the majority of both participant groups were from the US, and Western nations had considerably higher numbers of participants from each group. However, the population of nurses was more widely distributed around the world, compared to nursing students which were predominantly in the US and some European countries.

The main areas of practice and/or locations of care were medical‐surgical (*n* = 12), emergency room (*n* = 8), obstetrics/gynaecology (*n* = 6), ICU (*n* = 6), community health service centres (*n* = 5), acute care (*n* = 4), critical care (*n* = 3), paediatrics (*n* = 3), psychiatry (*n* = 2) orthopaedics (*n* = 2), neurology (*n* = 2), oncology (*n* = 1), geriatrics (*n* = 1), family practice (*n* = 1), outpatient clinics (*n* = 2), rehabilitation facilities (*n* = 2), primary care (*n* = 2), private nursing home (*n* = 1), bariatric centre of excellence (*n* = 1), university hospital (*n* = 1) and designated obesity facility (*n* = 1).

All studies included weight stigma or concepts related to it, or some aspect of it, including implicit or explicit attitudes and behaviours towards obesity, observed weight stigma, interventions addressing weight stigma, awareness of weight stigma, the impact of weight stigma on empathy, self‐reflection of weight stigma, behavioural intentions towards higher weight individuals, weight controllability beliefs, weight bias towards children or people with mental illness, attitudes towards personalities and lifestyles of higher weight individuals, perceptions of obesity, perceived causal factors of obesity, experiences of working with higher weight individuals, and ideological dilemmas in the perioperative care of higher weight individuals. Some studies included additional concepts such as internalised weight stigma, experienced weight stigma, body appreciation, quality of care, causal attributions for obesity, stigmatisation tendency, perceptions of provision of care, ethnic prejudices, empathy towards higher weight individuals and care decisions.

Studies covered a wide range of methodologies and study designs. Most studies were cross‐sectional (*n* = 25) and/or descriptive (*n* = 19). Eleven mixed‐methods studies (including one‐phase triangulation, partial, concurrent parallel) were found, in addition to qualitative studies (*n* = 8), quasi‐experimental studies (*n* = 6), correlational studies (*n* = 5), randomised controlled trials (RCT) (*n* = 3), one‐group repeated measures design (*n* = 3), randomised (*n* = 3), pre‐test/post‐test (*n* = 2), observational (*n* = 1), mixed design experiment (*n* = 1), methodological (*n* = 1), discursive psychology (*n* = 1) and cross‐cultural (*n* = 1) studies. Eight studies did not explicitly mention the study design, however, based on the explanation of methods and data collection instruments, the authors concluded these were all quantitative studies.

A variety of instruments were used across the included studies. Frequently used instruments included Attitudes Towards Obese Persons (ATOP, *n* = 22), Beliefs About Obese Persons (BAOP, *n* = 16), the Fat Phobia Scale (FPS, *n* = 14), Implicit Association Test (IAT, *n* = 9), Nurses' Attitudes Towards Obesity and Obese Patients Scale (NATOOPS, *n* = 9), the Anti‐Fat Attitudes (AFA, *n* = 4) and the Anti‐Fat Attitudes Test (AFAT, *n* = 2). Common qualitative data collection methods included individual interviews (*n* = 5), open‐ended questions (*n* = 5), journal entries (*n* = 2) and focus groups (*n* = 2). Vignettes were another popular tool used in multiple studies to evoke reactions towards higher weight individuals (*n* = 9).

### 4.2 The Nature of the Research

**FIGURE 4:**
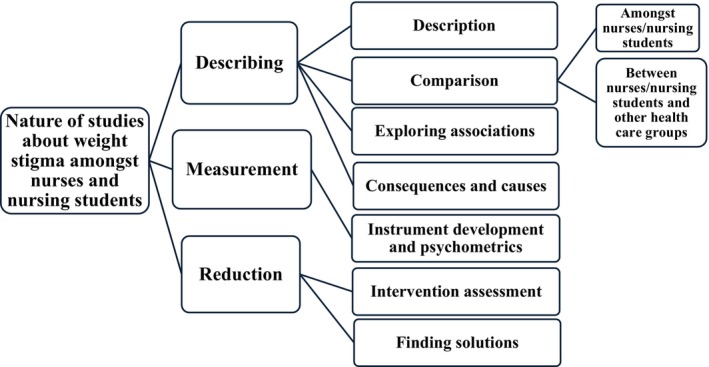
Diagram of categories of included studies based on the nature.

Weight‐stigma‐related objectives and key findings of the included studies were grouped into eight categories, including description, comparing different groups of nurses, exploring associations, intervention assessment, comparing nurses with other health professionals, exploring consequences or causes, instrument development and psychometrics and finding solutions. Further categorisation grouped these into three larger categories: describing, measurement and reduction (Figure 4).

**FIGURE 5:**
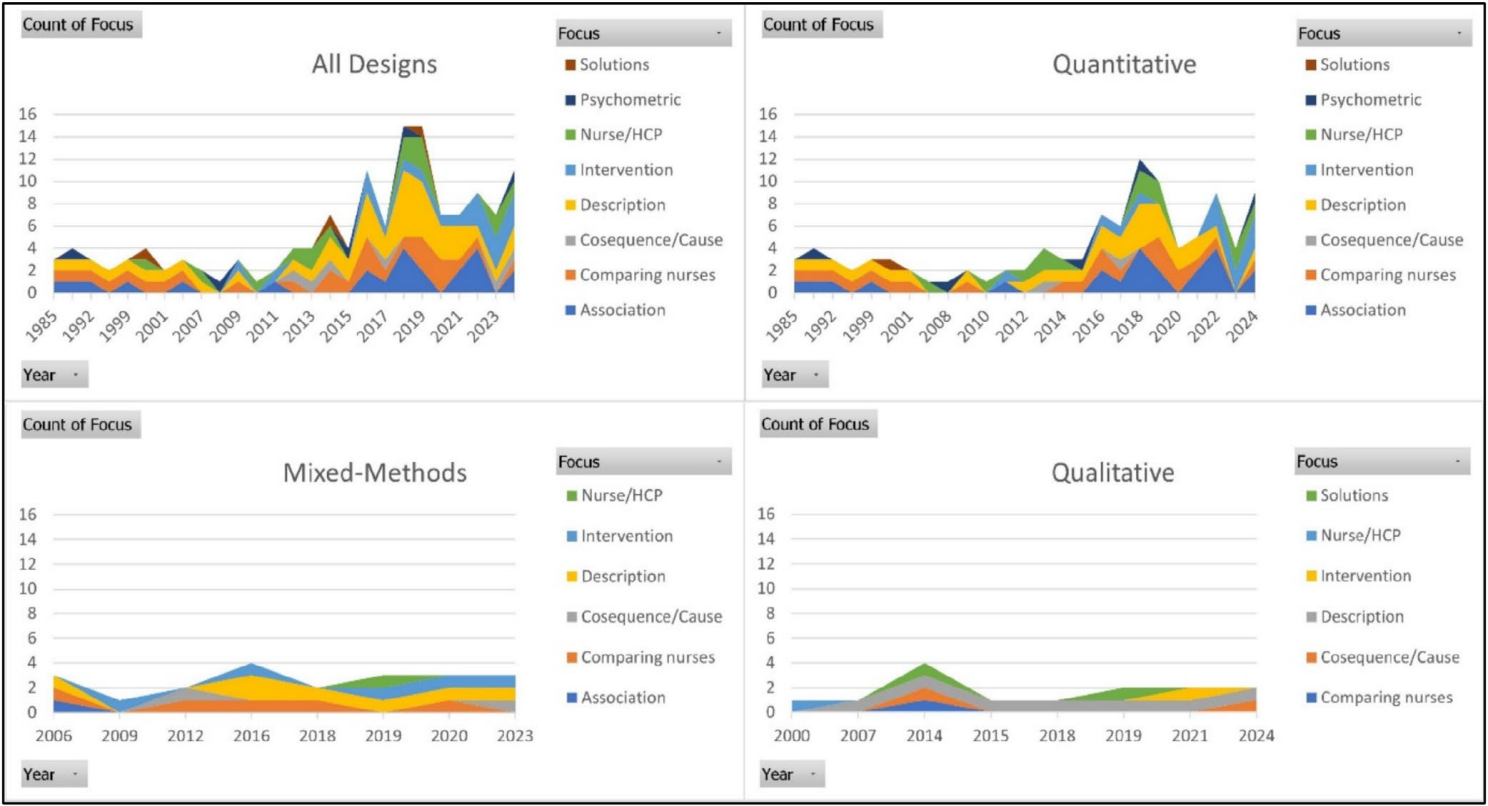
Specific areas of focus within studies over time. HCP, Health care professional.

**FIGURE 6:**
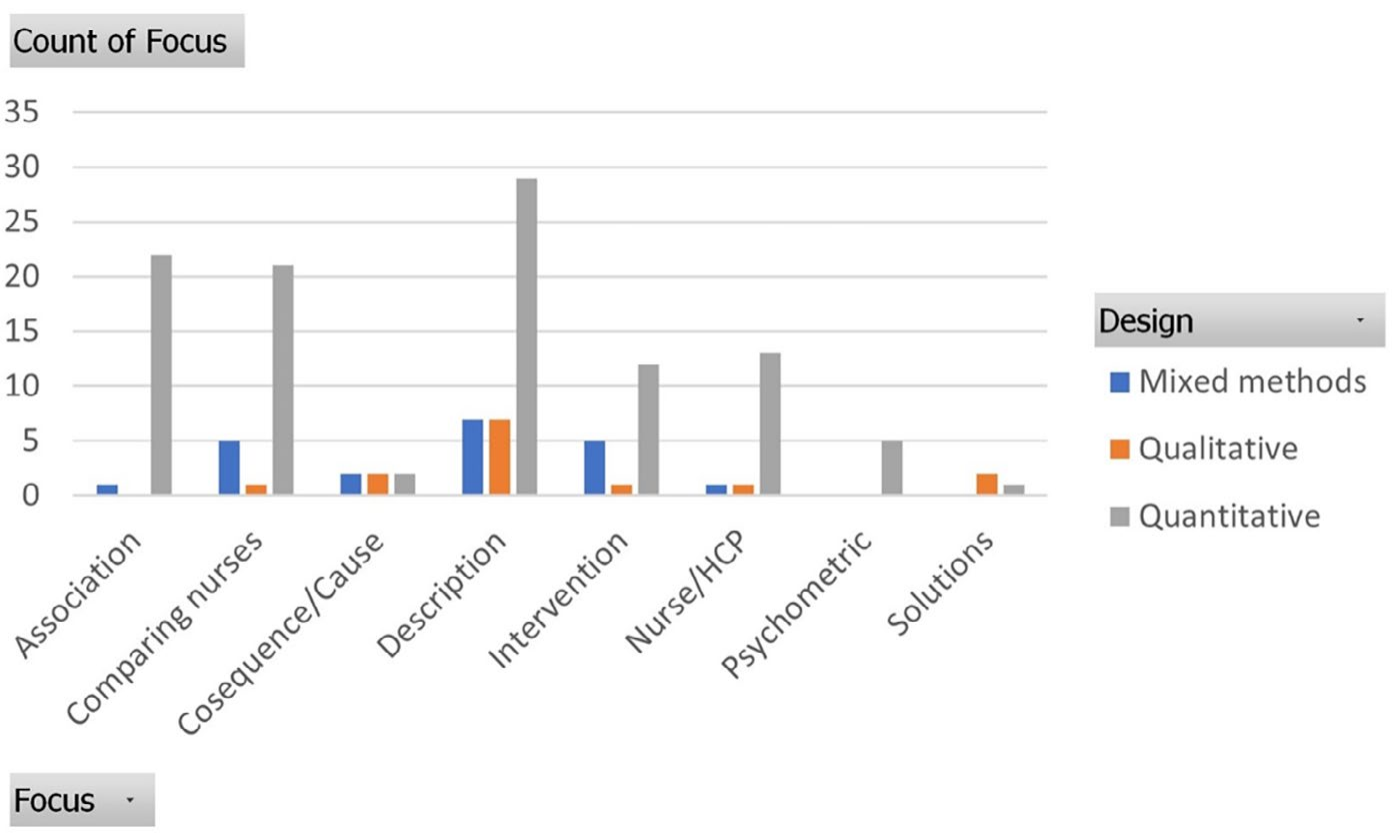
Areas of focus within studies by methodology. HCP, Health care professional.

Figures 5 and 6 will demonstrate the trend and prevalence of the nature of studies, respectively, in relation to their methodology.

As Figure 5 shows, across all methodologies, description was the most common nature of the study. Considering all designs combined, and quantitative studies, the prevalence of description was highest from 2016 to 2021. Whilst exploring weight stigma associations, comparing different groups of nurses and comparing nurses with other health professionals were the common natures of quantitative studies, finding solutions was mainly in qualitative studies. Comparing different groups of nurses and intervention assessment constitute a considerable section of the nature in mixed‐methods studies.

Figure 6 illustrates a notable proportion of quantitative studies across nearly all natures, except exploring consequences or causes of weight stigma, where there are equal numbers of corresponding studies from each methodology, and finding solutions, where qualitative methodology has been utilised more than quantitative.

All descriptors will be discussed in this section. Appendix S4 shows how each study contributes to the descriptors.

## 5 Descriptor 1: Description

More than half of included studies described weight stigma or aspects of it amongst different groups of nurses/nursing students, using various approaches (*n* = 43, Appendix S4).

### 5.1 Presence of Weight Stigma Amongst Nurses and Nursing Students

Multiple studies examined the presence and intensity of weight stigma amongst nurses/nursing students, frequently reporting negative attitudes within participants or observed by them, at different levels, even though positive attitudes were also identified (Table 1).

**TABLE 1:** Characteristics of weight stigma amongst nurses/nursing students.

Attitudes towards higher weight individuals	
Negative attitudes and/or beliefs towards higher weight individuals (*n* = 25)	(Bagley et al. 1989; Barra and Singh Hernandez 2018; Culbertson and Smolen 1999; Dunagan et al. 2016; Dunham 2024; Garner and Nicol 1998; George et al. 2019; Halvorson et al. 2019; Härgestam et al. 2024; Hauff et al. 2019; Johnson 2018; Kerbyson 2023; Maroney and Golub 1992; Mullaney 2016; Poon and Tarrant 2009; Robstad Siebler, Söderhamn, et al. 2018; Robstad, Söderhamn, Fegran, et al. 2018; Robstad et al. 2019; Tanner 2017; Usta et al. 2021; Waller et al. 2012; Ward‐Smith and Peterson 2016; Williams‐Hailey 2015; Yilmaz and Yabancı Ayhan 2018, 2019)
Negative attitudes and/or beliefs towards higher weight children (*n* = 2)	(Snethen et al. 2014; Thompson et al. 2021)
Positive attitudes towards higher weight individuals (*n* =7 )	(Geckle 2001; Lilliott 2000; Moyo 2022; Pfeiffer 2017; Salziyan et al. 2018; Wang et al. 2016; Zuzelo and Seminara 2006)

Observation of attitudes towards higher weight individuals	
Observing negative attitudes towards higher weight individuals (*n* = 5)	(Kerbyson 2023; Obitz and Frensborg 2014; Oliver et al. 2021a; Shea and Gagnon 2015; Thompson et al. 2021)
Observing warm weight‐bias‐free care by NP students from their preceptors for higher weight individuals (*n* = 1)	(Hauff et al. 2020)

Intensity of negative attitudes towards higher weight individuals	
Neutral attitudes towards higher weight individuals (*n* = 3)	(Gormley and Melby 2020; Young 1985; Zhu et al. 2013)
Low levels of negative attitudes towards higher weight individuals (*n* = 2)	(Mullaney 2016; Tanner 2017)
Slight implicit preference for thin bodies (*n* = 1)	(Dunham 2024)
Moderate levels of negative attitudes towards higher weight individuals (*n* = 3)	(Poon and Tarrant 2009; Usta et al. 2021; Yilmaz and Yabancı Ayhan 2019)
Moderate to strong implicit preference for thin bodies (*n* = 5)	(George et al. 2019; Halvorson et al. 2019; Robstad Siebler, Söderhamn, et al. 2018; Robstad et al. 2019; Waller et al. 2012)
High levels of negative attitudes towards higher weight individuals (*n* = 1)	(Yilmaz and Yabancı Ayhan 2018)

Developmental trend of attitudes towards higher weight individuals	
Undergraduate nursing students' negative attitudes towards higher weight individuals decreasing through academic years (*n* = 1)	(Rodríguez‐Gázquez et al. 2020)

### 5.2 Awareness of Weight Stigma

Acknowledgement of weight stigma by nurses/nursing students was reported in some studies (*n* = 5). Nurses were aware of weight bias, as well as environmental and social limitations associated with provision of care for higher weight individuals (Hauff et al. 2019), and lack of proper resources (Obitz and Frensborg 2014). Thirty per cent of undergraduate nursing students and recent graduates admitted to committing at least one fat‐phobic behaviour (Kerbyson 2023). Even though they observed weight stigma rooted in direct (such as provider's bias) or indirect factors (such as lack of skills, equipment/environment deficits or staffing issues), they mentioned that weight bias training is not necessarily compatible with the real‐world situation (Oliver et al. 2021a). Obesity was a sensitive topic for nurses to discuss with patients because they were aware of biases related to it and even the negative connotations attached to the word ‘obesity’; hence, they tried to avoid using it (Brown and Thompson 2007).

### 5.3 Manifestation of Weight Stigma

Multiple undesirable characteristics along with personal fault were attributed to higher weight individuals (*n* = 8). In terms of success, marriage suitability, tidiness, health, sociability and being good, NPs viewed higher weight individuals as inferior compared to others. Also, NPs perceived overeating and lack of physical activity as potential reasons for higher weight (Ward‐Smith and Peterson 2016). Other studies found that nurses believe higher weight individuals are lazy (Robstad Siebler, Söderhamn, et al. 2018; Robstad et al. 2019), non‐compliant, difficult to manage (Thompson et al. 2021), suspicious, weak‐willed (Williams‐Hailey 2015), undisciplined, unappealing (Tanner 2017), aggressive (Maroney and Golub 1992), untypical and resource‐demanding (Härgestam et al. 2024).

### 5.4 Internal Conflicts Regarding Weight Stigma

Some articles focused on the complicated aspects of internal conflicts experienced by nurses/nursing students regarding weight stigma (*n* = 8). Whilst working with higher weight individuals, nurses had ambivalent feelings towards them. These feelings emerged because nurses endeavoured to provide higher weight individuals with equal quality care as other patients, whilst simultaneously having negative beliefs and attitudes towards them. This made caring for higher weight individuals emotionally demanding for nurses (Robstad, Söderhamn, Fegran, et al. 2018), leading to overwhelming feelings because of the unique care needs (Zuzelo and Seminara 2006).

In other words, although nurses had the intention of immediately helping higher weight individuals (Robstad Siebler, Söderhamn, et al. 2018) and believed in the necessity of equitable care (Härgestam et al. 2024) and treating higher weight individuals with compassion and respect, they still held negative biases towards them (Thompson et al. 2021). Hiding prejudice against higher weight individuals, whilst being frustrated with them (Dunagan et al. 2016), made caring for higher weight individuals so difficult that nurses claimed they would avoid doing it if they had the option to do so (Maroney and Golub 1992). Also, nurses found working with higher weight individuals challenging due to observing weight bias in the words and behaviours of other nurses, along with previous experiences of negative attitudes towards higher weight individuals (Shea and Gagnon 2015).

## 6 Descriptor 2: Comparing Different Groups of Nurses

A category of studies compared different groups of nurses/nursing students in terms of weight stigma (*n* = 27, Appendix S4). The characteristics forming different groups were divided into demographic factors, educational and professional level, work setting and specialisation and cross‐cultural differences.

### 6.1 Demographic Factors

More than half of the studies in this descriptor compared nurses/nursing students of different demographics in terms of weight stigma (*n* = 17). Stronger negative attitudes were seen amongst White‐Caucasian nursing students (Johnson 2018), male nurses/nursing students (Johnson 2018; Robstad et al. 2019), female nursing students (Mullaney 2016; Usta et al. 2021), nursing students with extended families compared to those in nuclear families (Ozaydin and Tuncbeden 2022), older nurses (Bagley et al. 1989; Williams‐Hailey 2015), nursing students who count calories, and the ones with a dieting history (Usta et al. 2021).

On the other hand, more positive attitudes were seen amongst older nurses (Geckle 2001), nurses/nursing students with higher BMIs (Geckle 2001; Hemati and Zokaei 2016; Lilliott 2000; Usta et al. 2021; Yilmaz and Yabancı Ayhan 2019), nursing students with a higher weight family member (Yilmaz and Yabancı Ayhan 2019), female nursing students (Rodríguez‐Gázquez et al. 2020), Polish female nursing students compared to their male counterparts, and Nigerian male nursing students compared to their female counterparts (Styk et al. 2024).

Other studies found no significant difference in attitudes or feelings towards higher weight individuals between male and female nurses/nursing students (Garner and Nicol 1998; Gormley and Melby 2020; Hemati and Zokaei 2016; Obitz and Frensborg 2014), nurses of different weight statuses (Robstad et al. 2019; Young 1985) or nurses of different ages (Hemati and Zokaei 2016).

### 6.2 Educational and Professional Level

Some studies focused on comparing nurses/nursing students from different educational and professional levels (*n* = 12). More intense weight stigma was found amongst CNAs compared to RNs and LPNs (Garcia 2012), fourth‐year nursing students compared to the third‐year students (Ozaydin and Tuncbeden 2022), RNs compared to nursing students (Poon and Tarrant 2009; Yilmaz and Yabancı Ayhan 2019) and pre‐nursing compared to nursing students (Snethen et al. 2014).

More positive attitudes were identified amongst fourth‐year nursing students compared to those in their first year (Rodríguez‐Gázquez et al. 2020; Usta et al. 2021), and nurses with more years of professional education (Bagley et al. 1989).

Based on other studies, no significant difference was shown in weight stigma between nursing students of different academic years (Gormley and Melby 2020), between bachelor's and master's students (Culbertson and Smolen 1999), between nurses with different years of work experience (Hemati and Zokaei 2016), or between undergraduate and graduate nursing students in terms of their negative attitudes towards higher weight individuals and weight controllability beliefs (Darling and Atav 2019).

### 6.3 Work Setting and Specialisation

Some studies compared nurses from various work settings and specialisations (*n* = 4), showing higher weight stigma amongst nurses in medical/medical‐surgical units. Stronger weight stigma was found amongst medical centre RNs compared to acute rehabilitation nurses (Zuzelo and Seminara 2006), RNs in medical‐surgical units compared to those in specialist departments (Wang et al. 2016), and RNs in medical‐surgical areas of a community hospital who believed in more individually controllable factors contributing to higher weight, compared to nurses from a bariatric centre of excellence who expressed less negativity in terms of weight controllability attitudes (Hartman 2017).

However, three groups of NP students, from family practice, paediatrics and obstetrics/gynaecology fields did not differ significantly in relation to observed weight stigma (Hauff et al. 2020).

### 6.4 Cross‐Cultural Differences

Only a minority of studies compared nurses from different cultural backgrounds regarding their weight stigma (*n* = 2). Canadian nurses, compared to those in the US, expressed more weight controllability beliefs, repulsion at caring for higher weight individuals, and less empathy towards them (Maroney and Golub 1992). In another cross‐cultural study, Nigerian nursing students had more negative attitudes and beliefs towards higher weight individuals, compared to their Polish counterparts. However, Nigerian and Polish students' fatphobia scores were not significantly different (Styk et al. 2024).

## 7 Descriptor 3: Exploring Associations of Weight Stigma

Some studies explored whether nurses'/nursing students' weight stigma was associated with other factors (*n* = 23, Appendix S4).

### 7.1 Factors Positively Associated With Lower Weight Stigma

Based on some studies (*n* = 7), participants' favourable attitudes towards higher weight individuals were positively correlated with their own BMI (Gujral et al. 2011; Styk et al. 2024; Wang et al. 2016), self‐compassion (Joseph 2022), knowledge and favourable practices regarding higher weight (Moyo 2022), time spent on working with higher weight children (Thompson et al. 2021), and number of higher weight individuals in their daily care (Young 1985).

### 7.2 Factors Associated With Higher Weight Stigma

Other studies in this descriptor (*n* = 9) found that participants' weight bias was correlated with higher obesity prejudice (Ozaydin and Tuncbeden 2022), higher ethnic prejudice (Maroney and Golub 1992), negative attitudes towards caring for higher weight individuals (Bagley et al. 1989), less willingness to provide care for these patients (Usta et al. 2021), lower intentions to immediately help them (Robstad Siebler, Söderhamn, et al. 2018), lower age (Culbertson and Smolen 1999) and higher BMI (Styk et al. 2024). Nursing students' higher BMI was also associated with more experienced weight stigma in their healthcare history (Dunham 2024). Nurses' perception of higher weight individuals being treated differently in health care settings (by their colleagues or themselves) was significantly and positively associated with their weight controllability beliefs (Tanneberger and Ciupitu‐Plath 2018).

### 7.3 Non‐Significant Associations

Other studies (*n* = 11) found no significant correlation between nurses'/nursing students' weight stigma and their internalised weight stigma, experienced weight stigma, body appreciation (Hartman 2017), BMI (George et al. 2019; Ozaydin and Tuncbeden 2022; Salziyan et al. 2018; Zuzelo and Seminara 2006), age (Styk et al. 2024), previous experiences regarding higher weight history, personal or professional characteristics (Young 1985), nature of work setting, education level, years of work experience (Zuzelo and Seminara 2006), decision to prescribe pharmacotherapy for higher weight (Bottcher and Chao 2022), their advice given to higher weight individuals (Nicholls et al. 2016), behavioural intentions towards higher weight individuals (Robstad et al. 2019), and clinical decision‐making (Alexander 2018).

## 8 Descriptor 4: Intervention Assessment

A proportion of studies targeted nurses' or nursing students' weight bias, by conducting interventions and assessing the impacts on participants' weight bias (*n* = 18, Appendix S4). A summary of quantitative findings from this descriptor is provided in Table 2.

**TABLE 2:** Quantitative outcomes of interventions targeting weight stigma amongst nurses/nursing students.

Effective interventions	
Interventions improving beliefs about higher weight individuals and weight controllability (*n* = 5)	LEARN model and exposing participants to communicate with a higher weight individual in a simulation (Llewellyn et al. 2023) Case‐based Weight Bias Reduction (WBR) program (Oliver et al. 2022) Obesity sensitivity education (Marcum 2010) Bariatric sensitivity intervention (Molloy et al. 2016) CeWebs training (Oliver et al. 2020)
Interventions improving attitudes towards higher weight individuals (*n* = 6)	Bariatric sensitivity training (Gujral et al. 2011; Molloy et al. 2016) Obesity sensitivity program (Barra and Singh Hernandez 2018) Self‐directed obesity sensitivity education (Gamaly 2022) Interactive obesity sensitivity education (Gamaly 2022) CeWebs training (Oliver et al. 2020) Mindfulness‐based empathy training combined with obesity simulation suits (Can Gür and Yılmaz 2024)

Interventions with mixed or limited success	
Interventions (*n* = 2)	Targeted variables
Simulation‐based experiences with standardised participants, combined with WBR intervention (Oliver et al. 2024)	Beliefs and attitudes towards higher weight individuals (non‐significant improvement)
Educational modules on weight stigma and higher weight (Dunham 2024)	Implicit negative attitudes towards higher weight individuals (improvement in full‐time accelerated coursework track nursing students only)

Unsuccessful interventions for reducing weight stigma	
Interventions (*n* = 8)	Targeted variables
Bariatric sensitivity intervention (Gujral et al. 2011)	Beliefs about higher weight individuals
WBR intervention (Oliver et al. 2022)	Attitudes towards higher weight individuals
WBR intervention (Oliver et al. 2023)	Beliefs and attitudes towards higher weight individuals
Loving‐Kindness meditation (Joseph 2022)	Attitudes towards higher weight individuals
Obesity sensitivity education (Marcum 2010)	Attitudes towards higher weight individuals
Weight bias training (Gajewski 2023)	Empathy
An educational module for nursing students (Fruh et al. 2019)	Weight bias
The ‘Yale Rudd Centre Weight Bias in Healthcare’ video (Tanner 2017)	Fat‐phobia

### 8.1 Outcomes of Qualitative Investigations Following Interventions

Qualitative measurements (*n* = 5) following different interventions demonstrated greater awareness about personal (Fruh et al. 2019; Oliver et al. 2021b) and systemic weight stigma, greater ability to understand the impact of physical environments on weight stigma, and suggested education interventions for all health care providers (Fruh et al. 2019). These qualitative enquiries revealed awareness of the connection between insufficient resources and weight stigma, perception of higher weight as an involuntary condition (Oliver et al. 2021b), importance of patient‐centred care linked with listening and necessary modifications for higher weight individuals (Llewellyn et al. 2023), and less judgemental ideas towards higher weight individuals (Marcum 2010). Almost all nursing students reported being surprised after learning about the prevalence of weight stigma in health care and the difficulties attached to weight loss (Mullaney 2016).

## 9 Descriptor 5: Comparing Nurses With Other Health Professionals

One group of included studies focused on comparing nurses/nursing students with other health professionals or students in different fields, in terms of weight stigma (*n* = 15, Appendix S4).

### 9.1 Comparisons Showing Lower Weight Stigma Amongst Nurses/Nursing Students

#### 9.1.1 Compared to Physicians

Some studies (*n* = 4) found more favourable beliefs (Sikorski et al. 2013; Tuzun et al. 2023) and more positive attitudes towards higher weight individuals (Bucher Della Torre et al. 2018; Sikorski et al. 2013), lower perception of frustration and lower perception of weight bias by colleagues (van der Voorn et al. 2023) amongst nurses compared to physicians.

#### 9.1.2 Compared to Other Health Care Professionals

When compared to other health care professionals (*n* = 2), nurses had more favourable attitudes towards higher weight individuals compared to therapists and other medical providers (Sikorski et al. 2013), and lower fat‐phobia than physiotherapists (Wise et al. 2014).

#### 9.1.3 Student Comparisons

Studies involving students (*n* = 3) showed that nursing students had lower weight controllability beliefs than dietetics and medicine students, lower fat‐phobia than dietetics, nutrition (Swift et al. 2013) and medical students (Nickel et al. 2019; Petrich 2000; Swift et al. 2013), and greater empathy compared to medical students (Petrich 2000).

### 9.2 Comparisons Showing Higher Weight Stigma Amongst Nurses/Nursing Students

Some other studies (*n* = 4) found more negative attitudes and beliefs amongst nursing students compared to social work students (Darling and Atav 2019), higher implicit weight bias amongst paediatric nurses, compared to that of paediatric physicians and advanced practice providers (Turner 2024), more negative attitudes along with lower willingness for providing care to higher weight individuals amongst nurses compared to physicians (Akman et al. 2010), and more negative attitudes amongst nursing students compared to other health care employees (Frick 2007).

### 9.3 Comparisons Showing Mixed Findings

Mixed findings were shown in one study (*n* = 1). Wynn et al. (2018) reported relatively more favourable attitudes amongst nurses compared to operating department practitioners, pharmacists, junior doctors and consultants, and less favourable ones amongst nurses compared to dietitians, health care assistants and medical students. However, they did not report whether these differences was significant.

### 9.4 Comparisons Showing No Significant Differences

In terms of weight stigma related concepts, in some studies (*n* = 5), no significant difference was found between nurses or nursing students and psychologists (Tuzun et al. 2023), psychology students (Waller et al. 2012), paediatric hospitalists (Halvorson et al. 2019), advanced practice providers (Turner 2024), and physicians (Bucher Della Torre et al. 2018; Turner 2024; Tuzun et al. 2023).

## 10 Descriptor 6: Exploring Consequences Or Causes

A small group of studies explored the causes and consequences of weight stigma amongst nurses/nursing students (*n* = 6, Appendix S4).

Exploration of the causes of weight stigma from nurses' perspectives (*n* = 2) revealed both systemic and personal factors, including the tasks required to meet patients' needs, patients' characteristics, specific equipment requirements, nurses' self‐perception (Garcia 2012) and beliefs about whether or not higher weight is self‐inflicted (Obitz and Frensborg 2014).

Exploring consequences of weight stigma (*n* = 3) revealed the potential impact of observing colleagues' fat‐phobic behaviours on making the provision of bias‐free care much more challenging (Kerbyson 2023), the impact of perceiving higher weight individuals as untypical and resource‐demanding on ambivalent feelings and frustration whilst communicating with those patients (Härgestam et al. 2024), and the effect of weight bias on delaying the provision of treatment (Garcia 2012).

Other studies (*n* = 2) found areas that were not impacted by weight stigma and the patients' weight, including nurses' self‐efficacy or weight management practices (Zhu et al. 2013) and their decisions in relation to care, such as providing walk assists (Pfeiffer 2017).

## 11 Descriptor 7: Instrument Development and Psychometrics

Another small group of included studies focused on psychometric characteristics of tools to assess weight stigma amongst nurses (*n* = 5, Appendix S4).

### 11.1 Successfully Validated Instruments

Studies that focused on the assessment of behavioural intentions, NATOOPS and FPS questionnaire (*n* = 3) demonstrated acceptable to good psychometric characteristics. Designing and testing a set of instruments including IAT, AFA, explicit bias scale and vignettes for behavioural intentions assessment to measure ICU nurses' implicit and explicit attitudes towards higher weight individuals in Norway led to finding overall suitable validity and reliability (Robstad Siebler, Söderhamn, et al. 2018). Watson et al. (2008) developed and tested NATOOPS to specifically measure nurses' attitudes towards obesity and obese patients, the factors of which included response to higher weight individuals, characteristics of them, controllability of factors related to higher weight, stereotypic characteristics of higher weight individuals and supportive roles in caring for them. Satisfactory construct validity and Cronbach's alpha (0.81 for the scale, ranged 0.45 to 0.79 for the five subscales) were recorded. Styk et al. (2024) translated the FPS questionnaire into Polish and adapted it to the Polish population. Similar to the English version scales, a univariate factor structure accounting for 41% of the total variance was supported by their analyses. A Cronbach's alpha of 0.89 indicated the internal consistency of this new FPS version.

### 11.2 Other Instrument Development

From the remaining studies in this descriptor (*n* = 2), one study adapted existing instruments using content validity to develop an instrument measuring psychiatric nurses' attitudes, knowledge and behaviours towards higher weight individuals with mental illness. The subsections were knowledge, general attitudes towards obesity, intrinsic attitudes and self‐reported behaviours. The results did not show appropriate reliability for the instrument as a whole (Williams‐Hailey 2015). The other study used a pilot test which reduced a pool of 70 items to two shorter scales, measuring nurses' attitudes towards management of higher weight individuals, and their personality and lifestyle (Bagley et al. 1989).

## 12 Descriptor 8: Finding Solutions

The final category consisted of a minority of studies which asked nurses' or nursing students' opinions about the solutions to the issue of weight stigma (*n* = 3, Appendix S4). These studies revealed the importance of education for both nurses and patients to reduce weight bias in health care, especially to motivate higher weight individuals to seek care in the earlier stages of their diseases and to stop blaming themselves for their weight (Obitz and Frensborg 2014), the need for improving training programs, creating size‐inclusive equipment and offices, and redefining perceptions about obesity (Hauff et al. 2019), and the potential impact of sensitivity training on reducing weight bias (Lilliott 2000).

## 13 Discussion

Reviewing the literature about weight stigma amongst nurses and nursing students led to 80 studies, mostly published in the past decade, indicating an increased awareness about this field of research in recent years. Whilst many studies involved participants from the US, very few of them had been conducted in Australia, non‐Western countries and some European nations. The distribution of nursing students was even more concentrated in certain countries, compared to that of nurses, which was unexpected due to the reports of experienced weight stigma in health care from patients in various countries (O'Donoghue et al. 2021; Puhl et al. 2021; Ryan et al. 2023). The proportion of male, non‐binary and gender‐diverse participants was considerably lower than that of female ones. This was expected considering the higher proportion of female nurses shown in a 2020 survey in the US; however, the number of other genders is also slowly rising in the nursing workforce (Smiley et al. 2021). Therefore, more diversity in terms of nurses' cultural background and gender is recommended for future research.

Regarding the location of care, more studies had considered nurses working in medical‐surgical units, ICU, obstetrics/gynaecology areas and emergency room, compared to the ones focusing on paediatrics and family settings, geriatrics, psychiatry, neurology, oncology, orthopaedics, rehabilitation, primary care and even bariatric settings. This finding partly aligns with the considerable proportion of higher weight patients in ICU (Dennis and Trevenen 2016; Großschädl and Bauer 2022) and the weight stigma experienced by pregnant and postpartum women in health care (Incollingo Rodriguez et al. 2020). However, a stronger focus on other settings is also needed, especially considering the presence of weight bias in paediatric health professionals (Turner 2024), the association between cancer and obesity (Pati et al. 2023), musculoskeletal complications linked with obesity (Fortunato et al. 2021), and the critical role of primary care nurses in managing obesity (Brown and Thompson 2007).

In consideration of the nature of the study objectives and findings, studies were categorised using eight descriptors and then classified into three larger categories of describing, measurement and reduction, the diagram of which is shown in Figure 4. Based on the descriptors, most studies attempted to describe weight stigma amongst nurses or nursing students. The description was carried out by exploring the presence of stigmatising beliefs and attitudes amongst nurses/nursing students, nurses' awareness of weight stigma, their perception of higher weight individuals and the manifestation of their stigma whilst caring for those individuals in terms of feelings. Across all study designs, description was the most frequent nature of studies. On the other hand, only a minority of studies focused on finding solutions to the problem of weight stigma. This was the only nature where more qualitative designs were observed, compared to quantitative and mixed‐methods ones, which is consistent with their in‐depth purpose. However, there is a paucity of studies for finding solutions and studies with qualitative designs which should be addressed by future research.

Also, compared to the large number of studies focusing on factors correlated with weight stigma, only a few of them explored its causes and consequences in nursing care. Whilst the negative effect of health professionals' weight stigma on quality of care has been repeatedly shown by making higher weight individuals avoid or delay health care services (Alberga et al. 2019; Phelan et al. 2015a), the impact of nurses' weight stigma on the care provided by them (Garcia 2012; Pfeiffer 2017) was not adequately explored; this means examining the provided care by researchers, not from the patients' perspectives. Regarding the tools and instruments assessing nurses' weight stigma, multiple studies used nursing‐specific measurements, and some even developed such instruments or focused on their psychometric features. However, considering the cross‐cultural differences of nurses in their weight stigma (Maroney and Golub 1992; Styk et al. 2024), not enough studies considered adapting the tools to their participants' culture.

Overall, from 1985 to 2024, multiple studies indicated the presence of weight stigma amongst nurses/nursing students, discussed the factors associated with it, aimed to measure it and used various strategies to find solutions and decrease it. However, the issue of weight stigma still exists within the nursing practice. It has been recently shown that 30% of nurses and nursing students admit to doing at least one fat‐phobic behaviour (Kerbyson 2023). Also, narratives of higher weight women's experiences in health care and sometimes with nurses show that they feel less human and worthy than thinner women, struggle to fit in and are frequently dismissed (Merrill and Grassley 2008). Indubitably, nurses encounter obstacles whilst providing equal care which is free of weight bias (Shea and Gagnon 2015). Some included studies, mainly in the description category, discussed some of these unique challenges, including the specific care needs of those patients (Zuzelo and Seminara 2006), equipment requirements (Garcia 2012), lack of proper resources (Obitz and Frensborg 2014), stigma observed by colleagues (Shea and Gagnon 2015) and ambivalent feelings of nurses whilst caring for higher weight individuals (Härgestam et al. 2024; Robstad, Söderhamn, Fegran, et al. 2018). However, the challenges have not been specifically addressed by studies in this field. In addition, weight bias reduction training programs for nurses are not always suitable considering real‐world circumstances (Oliver et al. 2021a).

Multiple strategies have already been proposed to lower weight stigma in health care; such as including weight bias‐related modules in medical academic curricula to increase health professionals' self‐awareness of their own biases, teach them about complex underlying reasons of higher weight, and facilitate patient‐centred care (Puhl 2023). When paediatric health professionals, including nurses, were asked about their beliefs towards weight bias and interventions to reduce it, more than half of them supported continuing education courses, mandatory weight bias education in medical/nursing curricula, and anti‐bullying policies (Turner 2024). However, the development of nursing‐specific practical strategies to reduce weight stigma, especially through nursing academic curricula, and exploring nurses' experience of and attitudes towards them were not the focus of the included studies in this literature review. This is particularly important due to the nursing students' attitudes improvement towards higher weight individuals through their academic years (Rodríguez‐Gázquez et al. 2020; Usta et al. 2021), which is contrary to what medical students experience (Phelan et al. 2015b). Warm interactions observed by nursing students from their instructors (Hauff et al. 2020) might be partly responsible for this improvement. Hence, it is important to conduct nuanced explorations of changes in nursing students' attitudes and beliefs towards higher weight individuals in nursing schools, and the factors encouraging more positive attitudes.

Finally, an exploration of the state of current nursing academic curricula regarding weight and weight stigma is recommended, since nurse educators who develop programs were not amongst the main groups of participants in the included studies. Nurses are aware of their weight stigma, they try to avoid stigmatising language (Brown and Thompson 2007) and are willing to challenge their biases (Turner 2024). Therefore, preceptors can also provide nurses with practical strategies to take action towards dismantling weight stigma.

## 14 Limitations

Regarding limitations, this scoping review had to exclude non‐English studies. Additionally, judging whether nursing data could be extracted from studies involving populations from different health professions, might have led to missing some relevant data. Furthermore, possible reviewer bias whilst interpreting the findings might have caused some degree of inaccuracy.

## 15 Conclusion

This scoping review aimed to identify the extent, range and nature of both direct and comparative studies on weight stigma amongst nurses and nursing students. Results were provided based on 80 included studies, most of which had been conducted in the US, included significantly more female participants than other genders, and had a cross‐sectional and/or descriptive design. In terms of aims and key findings, most articles focused on weight stigma description amongst nurses or nursing students, comparison of multiple groups of nurses regarding their weight stigma, and exploration of associations between weight stigma and other factors. The results of intervention studies were inconclusive regarding weight stigma reduction. Findings demonstrated a need for further research with qualitative designs, and more diversity in participants' gender, location of care and countries, where the instruments are culturally adapted. Future research should also further concentrate on the causes of nurses' weight stigma and consequences for the provision of care, the development of attitudes through academic years, nurses' proposed solutions and developing practical strategies for stigma reduction. In addition, upcoming studies are required to address the barriers to and attitudes towards these strategies, the feasibility of modifying nursing academic curricula accordingly, and nursing program instructors' viewpoints on them.

## Author Contributions

Made substantial contributions to conception and design, or acquisition of data, or analysis and interpretation of data; M.F., B.E., A.T.B. Involved in drafting the manuscript or revising it critically for important intellectual content; M.F., B.E., A.T.B. Given final approval of the version to be published. Each author should have participated sufficiently in the work to take public responsibility for appropriate portions of the content; M.F., B.E., A.T.B. Agreed to be accountable for all aspects of the work in ensuring that questions related to the accuracy or integrity of any part of the work are appropriately investigated and resolved; M.F., B.E., A.T.B.

## Conflicts of Interest

The authors declare no conflicts of interest.

## Peer Review

The peer review history for this article is available at https://www.webofscience.com/api/gateway/wos/peer‐review/10.1111/jan.16843.

## Supporting information


Data S1.

Data S2.

## Data Availability

The data that supports the findings of this study are available in the Supporting Information of this article. In September 2023, a protocol for this scoping review was registered on the Open Science Framework website (https://doi.org/10.17605/OSF.IO/U8FPD).

## Endnotes

^1^
This protocol can be found in the following URL: https://doi.org/10.17605/OSF.IO/U8FPD.
