# Supplementary material for: Weight Stigma Amongst Nurses and Nursing Students: A Scoping Review of Direct and Comparative Evidence
Source: J Adv Nurs. 2025 Feb 24;81(9):5806–23. doi: 10.1111/jan.16843 (PMC12371829; doi:10.1111/jan.16843)
Supplement: Supplementary file 2 — Data S2. [file JAN-81-5806-s001.docx]

**Appendix 1: Search strategy**

MEDLINE- Search conducted: 23 August 2023

| **Search ID** | **Search terms** | **Retrieved results** |
| --- | --- | --- |
| S81 | S79 AND S80 | 388 |
| S80 | S44 OR S45 OR S46 OR S47 OR S48 OR S49 OR S50 OR S51 OR S52 OR S53 OR S54 OR S55 OR S56 OR S57 OR S58 OR S59 OR S60 OR S61 OR S62 OR S63 OR S64 OR S65 OR S66 OR S67 OR S68 OR S69 OR S70 OR S71 OR S72 OR S73 OR S74 OR S75 OR S76 OR S77 OR S78 | 1,705 |
| S79 | S1 OR S2 OR S3 OR S4 OR S5 OR S6 OR S7 OR S8 OR S9 OR S10 OR S11 OR S12 OR S13 OR S14 OR S15 OR S16 OR S17 OR S18 OR S19 OR S20 OR S21 OR S22 OR S23 OR S24 OR S25 OR S26 OR S27 OR S28 OR S29 OR S30 OR S31 OR S32 OR S33 OR S34 OR S35 OR S36 OR S37 OR S38 OR S39 OR S40 OR S41 OR S42 OR S43 | 1,600,397 |
| S78 | “obesity discrimination” | 213 |
| S77 | “anti-fat attitude*” | 112 |
| S76 | “overweight stigma” | 0 |
| S75 | “anti-overweight bias” | 0 |
| S74 | “fat prejudice” | 17 |
| S73 | “anti-obesity bias” | 5 |
| S72 | “obesity stigma” | 125 |
| S71 | “weight-based discrimination” | 53 |
| S70 | “stigma of overweight” | 10 |
| S69 | “fat sham*” | 36 |
| S68 | Fatphobia | 200 |
| S67 | “Fat-phobia” | 283 |
| S66 | “overweight discrimination” | 2 |
| S65 | “overweight prejudice” | 0 |
| S64 | “anti-overweight prejudice” | 0 |
| S63 | “anti-overweight attitude*” | 0 |
| S62 | “overweight bias” | 196 |
| S61 | “fat discrimination” | 14 |
| S60 | “anti-fat prejudice” | 16 |
| S59 | “anti-obesity attitude*” | 2 |
| S58 | “anti-fat bias” | 234 |
| S57 | “anti-obesity prejudice” | 1 |
| S56 | “obesity prejudice” | 9 |
| S55 | “stigma of obesity” | 71 |
| S54 | “obesity bias” | 248 |
| S53 | “weight-related discrimination” | 18 |
| S52 | “weight discrimination” | 379 |
| S51 | “stigma of weight” | 86 |
| S50 | “stigmatization of overweight” | 16 |
| S49 | “stigmatization of weight” | 5 |
| S48 | “weight stigmatization” | 66 |
| S47 | "weight prejudice" | 208 |
| S46 | (MH "Weight Prejudice") | 198 |
| S45 | “weight stigma” | 715 |
| S44 | “Weight bias” | 621 |
| S43 | “healthcare provider*” | 68,887 |
| S42 | “healthcare student*” | 1,079 |
| S41 | “healthcare specialist*” | 202 |
| S40 | “practitioner* in healthcare” | 20 |
| S39 | “healthcare expert*” | 208 |
| S38 | “healthcare worker*” | 54,338 |
| S37 | “healthcare practitioner*” | 2,702 |
| S36 | “healthcare personnel” | 2,964 |
| S35 | “Healthcare professional*” | 39,627 |
| S34 | “health care student*” | 530 |
| S33 | “medical undergraduate*” | 27,913 |
| S32 | “medical candidate*” | 32 |
| S31 | “medical learner*” | 322 |
| S30 | “medical apprentice*” | 19 |
| S29 | “medical intern*” | 9,217 |
| S28 | “medical student*” | 69,542 |
| S27 | “medical scholar*” | 246 |
| S26 | “medical trainee*” | 1,792 |
| S25 | “health service professional*” | 94 |
| S24 | “clinical practitioner*” | 1,325 |
| S23 | “medical practitioner*” | 7,509 |
| S22 | “medical expert*” | 5,193 |
| S21 | “health care specialist*” | 226 |
| S20 | “caregiver* in health” | 20 |
| S19 | “medical personnel” | 7,509 |
| S18 | “health worker*” | 27,763 |
| S17 | “practitioner* in health care” | 24 |
| S16 | “health care expert*” | 178 |
| S15 | “clinical personnel” | 372 |
| S14 | “health care worker*” | 18,813 |
| S13 | "medical staff" | 41,921 |
| S12 | (MH "Medical Staff") | 3,003 |
| S11 | “health care provider*” | 77,497 |
| S10 | “care provider*” | 70,922 |
| S9 | “clinical professional*” | 604 |
| S8 | “health care practitioner*” | 2,888 |
| S7 | “medical professional*” | 12,435 |
| S6 | “health care personnel” | 3,119 |
| S5 | “health professional*” | 71,971 |
| S4 | "health personnel" | 208,390 |
| S3 | (MH "Health Personnel") | 64,267 |
| S2 | “Health care professional*” | 32,332 |
| S1 | nurs* | 1,148,581 |

**Appendix 2: Logic grid of the scoping review**

| **Population** | **Concept** | **Context** |
| --- | --- | --- |
| Nurs* | “Weight bias” |  |
| “Health care professional*” | “weight prejudice” |  |
| “health professional*” | “weight stigma” |  |
| “health care personnel” | “weight discrimination” |  |
| “health personnel” | “obesity bias” |  |
| “medical professional*” | “obesity stigma” |  |
| “health care practitioner*” | “stigma of obesity” |  |
| “clinical professional*” | “obesity discrimination” |  |
| “care provider*” | “obesity prejudice” |  |
| “health care provider*” | “anti-obesity prejudice” |  |
| “health care worker*” | “anti-fat bias” |  |
| “medical staff” | “anti-fat attitude*” |  |
| “clinical personnel” | “anti-obesity bias” |  |
| “health care expert*” | “anti-obesity attitude*” |  |
| “practitioner* in health care” | “anti-fat prejudice” |  |
| “health worker*” | “fat prejudice” |  |
| “medical personnel” | “fat discrimination” |  |
| “caregiver* in health” | “overweight bias” |  |
| “health care specialist*” | “overweight stigma” |  |
| “medical expert*” | “anti-overweight bias” |  |
| “medical practitioner*” | “anti-overweight attitude*” |  |
| “clinical practitioner*” | “anti-overweight prejudice” |  |
| “health service professional*” | “overweight prejudice” |  |
| “medical trainee*” | “overweight discrimination” |  |
| “medical scholar*” | “Fat-phobia” |  |
| “medical student*” | Fatphobia |  |
| “medical intern*” | “fat sham*” |  |
| “medical apprentice*” | “weight-based discrimination” |  |
| “medical learner*” | “weight-related discrimination” |  |
| “medical candidate*” | “weight stigmatization” |  |
| “medical undergraduate*” | “stigmatization of weight” |  |
| “health care student*” | “stigmatization of overweight” |  |
| “Healthcare professional*” | “stigma of weight” |  |
| “healthcare personnel” | “stigma of overweight” |  |
| “healthcare practitioner*” |  |  |
| “healthcare provider*” |  |  |
| “healthcare worker*” |  |  |
| “healthcare expert*” |  |  |
| “practitioner* in healthcare” |  |  |
| “healthcare specialist*” |  |  |
| “healthcare student*” |  |  |

**Appendix 3: The results of included studies**

| # | Author (year) | Publication type/ source | Country | Participants: sample size, gender, qualification, field of study/practice | Study aims/ objectives | Concept | Location of care | Type of study: design, methodology | Data collection instruments (weight stigma related) and interventions (if applicable) | Relevant key findings |
| --- | --- | --- | --- | --- | --- | --- | --- | --- | --- | --- |
| 1 | Akman et al. (2010) | JA | Turkey | 104 HCPs, including 51 nurses (51F) | To investigate attitudes and professional practice patterns of primary health care providers towards obese patients | Weight bias | Primary care | Cross-sectional survey | OPS, subtopics: personal attitudes and professional practice patterns | More negative attitudes among nurses compared to physicians (for total OPS score and both subtopics). Significantly more nurses than physicians did not want to work with an obese person, they preferred not to provide service to an obese person, they believed that obese patients always regained the weight they lost, obese patients failed to engage in their daily tasks, and they were not on time for their appointments. |
| 2 | Alexander (2018) | D | USA | 417 prelicensure nursing students (370F) | To examine if implicit and explicit bias scores were associated with decision making | Weight bias | _ | An experimental design with randomization | Vignettes – Computer-based IAT – Short form of FPS | Low to no linear relationship between participants’ weight bias and decision making. |
| 3 | Bagley (1989) | JA | Canada | 107 graduate RNs (107F) | To develop a scale to measure nurses' attitudes about obese patients | Attitudes about obese patients | Three urban hospitals | Quantitative | A 15-item Nursing Management Scale and a 13-item Personality and Lifestyle Scale | A negative perception of obese adults is linked to a negative view of caring for obese patients. Less favourable views of obesity and obese patients among older nurses. More favourable attitudes among those with more years of professional education. Relationship between dissatisfaction with own body weight and negative attitudes toward obese adults. 24.3% and 12.1% agreed or strongly agreed with ‘caring for an obese patient usually repulses me’, and ‘I'd rather not touch an obese patient’, respectively. |
| 4 | Barra & Singh Hernandez (2018) | JA | USA | 103 nursing students engaging in a medical surgical clinical practicum | To determine the efficacy of an obesity sensitivity program to determine nursing students’ attitudes toward obese clients | Weight bias | _ | Quantitative | Obesity sensitivity program (intervention) - ATOPS - Three series of vignettes | Pre-project: negative opinions about obese patients along with concerns regarding back injuries, among more than half of the students. A significant positive change in weight prejudices post-obesity education intervention, among all clinical groups. |
| 5 | Bottcher & Chao (2022) | JA | USA | 74 adult or family NPs (69F) | To examine the relationship between weight bias and prescription of obesity pharmacotherapy by NPs – To evaluate if providers’ views of the causal attributions of obesity are associated with the likelihood of prescribing weight management pharmacotherapy | Stigmatizing attitudes toward obesity - Causal Attributions for Obesity | Solo/group private practice – Hospital/medical centre - Public clinic/community health centre - University health system - Practice network/health maintenance organization | Cross-sectional design | Practices concerning obesity pharmacotherapy – CAO scale - NATOOPS | Prescription of obesity pharmacotherapy by 17.6%. No significant difference in beliefs about the controllability of obesity or weight bias among the two groups. |
| 6 | Brown & Thompson (2007) | JA | UK | 15 RNs (15F) with substantial experience of working in primary care: Practice nurses (9) - District nursing staff (4) - Health visitors (2) | To explore primary care nurses’ attitudes and beliefs in relation to giving patients advice about obesity | Awareness of obesity stigma | Primary care | Pragmatic qualitative methodology | Individual interviews | Awareness of obesity stigma (social judgements) and negative psychological and health impacts related to obesity among participants, leading to the perception of obesity as being a sensitive issue to discuss (one example: avoiding the term ‘obesity’, because of its negative connotations). |
| 7 | Bucher Della Torre et al. (2018) | JA | Switzerland | 834 HCPs, including 502 nurses | To assess HCPs’ attitudes and beliefs about obesity – To compare these outcomes between physicians and nurses | Attitudes and beliefs about obesity | _ | Quantitative | A developed questionnaire to explore attitudes towards obesity and patients with obesity (in addition to other factors) | Higher score for beliefs regarding genetic and endocrinological factors causing obesity, among nurses. No difference between physicians and nurses for beliefs regarding behavioural factors causing obesity. Slightly more negative attitudes among physicians, compared to nurses. |
| 8 | Can Gür & Yılmaz (2024) | JA | Turkey | 84 nursing students (86.9%F) | To investigate the impact of mindfulness-based empathy training and obese simulation suits on nursing students' attitudes and empathy levels towards obese patients | Attitudes and empathy towards obese patients | _ | RCT with a pre-test, post-test, and repeated measurement design and a control group | NATOOPS – Turkish adaptation of JSENS – MBET-OSS (intervention) | Statistically significant improvement in attitudes and empathy levels among experimental group, compared to the control group; as well as in post-test and follow-up scores of the experimental group’s empathy and attitudes, compared to the pre-test setting. |
| 9 | Culbertson & Smolen (1999) | JA | USA | 73 RN students (66F), including 33 bachelor and 40 master’s students) | To explore attitudes of RN students toward personalities and lifestyles and management of nursing care of obese adult patients – To explore effects of RN demographic variables on their attitudes – To explore if there is a relationship between attitudes toward personalities/lifestyles and attitudes toward the management of nursing care | Attitudes toward personalities and lifestyles and management of nursing care of obese adult patients | _ | Descriptive correlational survey design | Nurses’ Attitudes Toward Obese Adult Patients (measuring attitudes toward personalities and lifestyles of obese adults, and attitudes toward management of care) | Negative attitudes of RNs toward personalities and lifestyles of obese patients (the majority agreed on weight controllability and poor food selection) and management of care (perceiving it as physically exhausting being uncomfortable while doing it, and preference to avoid doing it if given a choice). No significant difference in bachelor and master’s students’ attitudes toward both lifestyles/personalities and management of care. Negative correlation between age and negative attitudes toward both lifestyles/personalities and management of care. Strong positive relationship between the two groups of attitudes. |
| 10 | Darling & Atav (2019) | JA | USA | Undergraduate and graduate nursing students (n=338), and graduate education and social work students | To assess the attitudes of graduate and undergraduate students toward the obese population _ To compare nursing student attitudes with those of students in other professional fields | Attitudes toward obese people | _ | A non-experimental ex post facto, cross-sectional design | ATOP – BAOS | Significantly more negative attitudes and negative controllability beliefs among nurses, compared to social work students. No difference between attitudes and controllability beliefs of undergraduate and graduate nursing students. |
| 11 | Dunagan et al. (2016) | JA | USA | 50 baccalaureate nursing students (86%F) | To describe attitudes of prejudice reported by baccalaureate nursing students | Attitudes of prejudice (including attitudes of prejudice about obese patients) | _ | A one-phase triangulation mixed-methods design (only reports of qualitative data in this article) | An open-ended question focused on nursing students’ experiences of having attitudes of prejudice, via a Web-based online survey | Two major themes, one of which being ‘prejudice against obese individuals’, when students were frustrated with obese people, yet tried not to express their bias even when they perceived patients would or did not care for themselves. Recalling an experience working as a paramedic when a “lesser quality of care was provided” due to prejudice against an obese patient, by a participant. |
| 12 | Dunham (2024) | JA | USA | 139 nursing students, including 26 juniors, 24 seniors, and 89 FACT students (94 and 64 students completed pre- and post-test IAT, respectively) | To determine if obesity bias awareness impacts obesity bias – To determine the association between BMI and experienced obesity bias in healthcare | Implicit obesity bias – Experienced obesity bias | _ | Quantitative quasi-experimental | The Harvard Weight IAT – One question about experiencing obesity bias in healthcare, using a 5-point Likert scale - Three education modules on obesity and obesity bias in healthcare | Slight bias against obesity in both pre- and post-education settings. Lower post-education bias, compared to pre-education, but no statistically significant difference. Significant decrease in post-education obesity bias among FACT nursing students. Higher scores of experienced obesity stigma among students with a higher BMI. |
| 13 | Frick (2007) | D | USA | 20 undergraduate nursing students, and 17 other health care employees | To evaluate the effect of participants’ profession on pre-test bias vs. post-test bias vs. four-week follow-up bias | An intervention addressing anti-fat bias | _ | A pre-test/post-test/follow-up assessment | Intervention: a one-hour HAES-based educational in-service – AFAT | Increase in composite scores of undergraduate nursing students by 36%, 23%, and 30%, in beliefs/emotions, perceptions, and attributions constructs of AFAT, respectively (compared to other participants in other professions). |
| 14 | Fruh et al. (2019) | JA | USA | Pre-test 45, post-test 43, all female, students enrolled in a Women’s Health NP concentration | To examine the impact of implementing educational modules to determine if they improve knowledge and comfort levels for NP students (including weight bias) | Comfort and knowledge in obesity management (including bias towards obese patients) | _ | Educational intervention study, quantitative and qualitative | Qualitative and quantitative material designed for this study | Quantitative: no changes in weight bias from pre-test to post-test. Qualitative: increased awareness on personal and environmental weight bias, easier identification of contribution of physical environment to weight stigma, suggesting education about weight bias for all employees. |
| 15 | Gajewski (2023) | JA | USA | 121 undergraduate nursing students (101F) in their first semester of the nursing program (70 students from the traditional cohort class and 51 from the accelerated second-degree) | To identify the efficacy of weight bias training on the empathy skills of student nurses | Impact of weight bias training on empathy | _ | A quasi-experimental design | Learning activities on weight bias - A simulation activity with a standardized patient wearing an obesity suit - JSE-HPS – No measurements of weight bias | No significant difference in empathy scores before learning activities and post-learning activities. Significant increase in empathy scores in the accelerated second-degree cohort. No significant change in empathy scores of the traditional cohort class. |
| 16 | Gamaly (2022) | D | USA | 80 RNs (91.25%F), providing direct patient care to an adult population | To compare the effectiveness of two online learning methods (self-directed and interactive) to reduce obesity bias | Obesity bias | Clinical practice in the hospital setting | A pre-test/post-test/30-day post-test design | Intervention: self-directed or interactive obesity sensitivity education (both online) – ATOP | No difference between self-directed and interactive online education on decreasing obesity bias among nurses in a hospital setting. Statistically significant reduction in weight bias of both groups, from the pre-test to post-test number 2. |
| 17 | Garcia (2012) | D | USA | Quant: 113 nurses (94F): associates (51), bachelors (21), or master’s (6) degree, LPN (10), CNA (4) / Qual: 16 nurses (15F) | To determine if weight bias existed – To explore its relationship with BMI – To identify its causes – To evaluate its effects on quality of care | Weight bias – Quality of care | Quant: Medical/surgical (27) – emergency – intensive (20) – surgery (14) – obstetrics (9) – IV therapy/other (3) / Qual: 3, 4, 4, 2, 1, 1, respectively | Partial mixed-methods | Web-based version of NATOOPS – Semi-structured interview | Higher chance of under/normal weight nurses to have weight bias, and overweight/obese nurses to associate negative characteristics to obese patients. CNAs exhibiting more bias than RNs and LPNs. Themes for causes of weight bias: patient care tasks; characteristics of the patient; equipment needs; nurse perception of self. Impact of delays in treatment on quality of care. |
| 18 | Garner & Nicol (1998) | JA | USA | 68 nurses (45F) | To explore nursing staff’s attitudes toward obese clients and compare male and female nurses’ attitudes toward obesity | Attitudes toward obese clients | _ | Quantitative | ATOP | Negative attitudes toward obese clients among 25% of nurses. No sex differences in reported negative feelings of nurses; except this item ‘bathing obese clients repulses me’, to which 18% of women responded negatively whereas none of the men did. |
| 19 | Geckle (2001) | MT | USA | 134 participants (129F, 4M, 1 unmarked), including 111 RNs & 22 LPNs (1 not identified licensure status) | To examine attitudes of nurses toward obese adult patients | Attitudes toward obese adult patients | A non-profit metropolitan hospital (Emergency, Obstetrics, Oncology, Medical-Surgical, Orthopaedics, Neurology, and Intensive-Coronary Care Units) | A non-experimental, descriptive design | ANTOAP | Slightly positive overall attitude toward obese adult patients. More positive attitudes toward obese adult patients among older nurses (not significant), and overweight ones (significant). No statistical significance between nurses’ number of years of practice or years of nurses’ education and their attitudes toward obese adult patients. |
| 20 | George et al. (2019) | JA | USA | 69 (59F) nursing students (first semester junior bachelor of science in nursing) | To determine whether nursing students’ explicit beliefs about weight preference were consistent with the implicit attitudes from the IAT – To determine if weight bias is associated with the individual’s own weight category | Unconscious Weight Bias | _ | A descriptive study | The Harvard Weight IAT - Self-reported weight preference/bias | IAT results: 50 preference of thin, 13 no preference, 6 preference of fat. Exhibiting more weight-related bias on the IAT compared to their self-reported preference, by the majority of participants. No association between BMI and weight preference towards others. |
| 21 | Gormley & Melby (2020) | JA | UK | 211 students on Adult and Mental Health nursing courses (187F) | To ascertain nursing students' attitudes towards obese and overweight people | Attitudes towards obese and overweight people | _ | A descriptive correlational design | ATOP | Neutral attitudes towards overweight and obese people. No significant difference in the ATOP between Adult and Mental Health students, between males and females, or across year groups. |
| 22 | Gujral et al. (2011) | JA | USA | 266 RNs, caring for adults in various units, including bariatric patients | To determine if bariatric sensitivity training improves nursing attitudes and beliefs towards obese patients – To explore if nurses’ BMI impact their attitudes and beliefs | Attitudes and beliefs towards obese patient | Medical, emergency, obstetrics/gynaecology, and surgical units | Comparing two groups, one of which has received bariatric sensitivity training (intervention) and the other has not (control), with an on-line survey | Bariatric sensitivity training - ATOP - BAOP | Potential of annual bariatric sensitivity training in improving nursing attitudes, but not beliefs, towards obese patients. Marginal association of higher nurses’ BMI with more favourable attitudes and beliefs towards obese patients. |
| 23 | Halvorson et al. (2019) | JA | USA | 52 participants, including 8 nurses (8F) | Quantitative: to quantify implicit and explicit weight bias of paediatric inpatient providers | Implicit and explicit weight bias | Paediatric acute care | A mixed-methods study | IAT – AFA | Quantitative: moderate or strong preference for thin people among 50% of nurses. No significant difference between paediatric nurses and paediatric hospitalists, in terms of their implicit and explicit weight bias scores. |
| 24 | Härgestam et al. (2024) | JA | Sweden | 15 RNAs (6F), experienced in caring for obese patients in surgical procedures | To analyse how RNAs position themselves toward obese patients in perioperative care, while striving to provide equitable care | Ideological dilemmas in perioperative care of obese patients | A university hospital | Discursive psychology | Semi-structured focus group interviews, using vignettes | Description of obese patients as “untypical” and “resource-demanding” by RNAs, leading to conflicting feelings and frustration while communicating with them; despite RNAs mentioning the importance of equal treatment and care for obese patients in perioperative settings. |
| 25 | Hartman (2017) | D | USA | 117 RNs (104F) | To compare RNs attitudes towards patients with high BMI in a Bariatric Centre of Excellence and those in a community hospital – To determine if a relationship exists between nurses’ attitudes towards higher weight patients and their own body satisfaction, internalized weight bias, and experiences with weight stigmatization | Attitudes towards high BMI patients – Internalized weight stigma – Experienced weight stigma – Body appreciation | Medical-surgical areas, in a community hospital and a bariatric centre of excellence | Quasi-experimental design, using a comparative descriptive survey | NATOOPS – WBIS-M – Brief version of SSI – Body Appreciation Scale | No significant difference between two hospitals, regarding total score of NATOOPS and all of its subsections, except ‘controllable factors contributing to obesity’ (higher among nurses in the community hospital). No significant correlations between nurses’ attitudes toward higher weight individuals and their internalized weight stigma, experienced weight stigma, and their own body appreciation. |
| 26 | Hauff et al. (2019) | JA | USA | 44 women’s health NP students (all women, mostly RNs) | To explore NP students’ (1) encounters with obesity stigma and bias in their clinical environment and (2) recommendations to decrease obesity stigma and bias | Witnessed obesity bias | Acute and/or ambulatory care obstetrics/gynaecology facilities | Descriptive qualitative study | An online-based NP education program and an accompanying assignment, followed by creating a brief initial post and then, reflecting on another student’s initial post | Themes for objective 1: issues in the built and social environment, awareness of obesity bias, perceptions of individuals with larger bodies. Themes for objective 2: improving training programs, creating size inclusive office spaces and equipment, redefining perceptions of obesity. |
| 27 | Hauff et al. (2020) | JA | USA | 225 NP students, 89.3%/F (RNs enrolled in an online graduate-level Family NP) | To explore how NP students perceive preceptors’ behaviours when managing patients with obesity | Observed weight stigma | Family practice – Paediatrics - Obstetrics/ Gynaecology | Exploratory, cross-sectional study, using a concurrent parallel mixed-method approach | Observed preceptors’ interactions with patients with obesity - Qualitative feedback on preceptor interactions and observations of bias | No statistically significant difference between the groups on observing weight bias. Frequency of “Not Person-First Language” across the 3 groups. Observing preceptors displaying interpersonal warmth while working with obese patients, without weight bias. |
| 28 | Hemati & Zokaei (2016) | JA | Iran | 80 nurses (62F) | To explore attitudes and beliefs of nurse about excessive body weight and obesity | Attitudes and beliefs about excessive body weight and obesity | _ | A descriptive cross-sectional survey | ATOP – BAOP – An open-ended question (for additional revelation of attitudes and beliefs) | No significant difference based on age, gender, and work experience of nurses and their attitudes toward or beliefs about obese people. Significantly more positive attitudes toward and beliefs about obese people by overweight (rather than underweight or normal weight) nurses. |
| 29 | Johnson (2018) | D | USA | Quant: 116 (100F, 16M) BSN first-year (52) and final-semester students (64) / Qual: 5 (3 first-year, 2 final-semester) | To understand the attributions made by BSN students about the cause of obesity, resulting in weight bias toward obese patients | Weight bias negative attitudes toward obesity | _ | Mixed-methods study | Quant: NATOOPS / Qual: focus group interviews | Negative attitudes toward obesity among student nurses – Higher scores of the factors ‘response to obese patients’ among final-semester students and ‘supportive roles for caring for obese patients’ among 1^st^ year students – Stronger negative attitudes among males and White-Caucasian students, but no difference in BMI categories. Themes for perception of obese patients: self-indulgent, lacking self-control, and being more challenging to care for. |
| 30 | Joseph (2022) | D | USA | 189 student nurses (181F, 7M, 2TG, 1NB) | To explore the feasibility of the LKM as a brief intervention to reduce weight bias – To explore if self-compassion is associated with weight bias | Weight bias | _ | RCT | LKM intervention – ATOP – IAT | No significant difference between two groups, in terms of weight bias (ATOP or IAT scores). Positive correlation between positive attitudes towards people with obesity and self-compassion. |
| 31 | Kerbyson (2023) | HT | USA | 67 undergraduate nursing students and recent graduates (62F, 57 students) | To investigate the incidence of fat-phobic behaviours among the healthcare team and how nursing students’ and recent graduates’ observations of fat-phobic behaviours impact the provision of affirming care | Fat-phobic behaviours – Observed fat-phobic behaviours – Impact of Observed fat-phobic behaviours on provision of affirming care | _ | Cross-sectional study with a mixed-methods approach | FPS - Both quantitative and qualitative questions about instances of observed fat-phobia | Self-reported engaging in at least one of the fat-phobic behaviours, among 30%. Reporting observing another nurse or nursing assistant engage in at least one fat-phobic behaviour, among 34%. 31% either “agree” or “strongly agree” that such behaviours shown by others have made it considerably more demanding for them to provide affirming care. Themes: 1) Observations of Fat-phobia (disrespectful language and actions, weight loss suggestions), 2) Discomfort Caring for Larger Patients (emotional distress, fear of injury, lack of support), 3) Impact of Clinical Experiences (Negative Impact) |
| 32 | Lilliott (2000) | MT | USA | 137 RNs | To determine if RNs had favourable or unfavourable attitudes toward obese adult patients – To determine the relationship of a nurse’s attitude to variables such as the nurse’s BMI and level of education – To ask nurses for suggestions to positively improve their attitudes toward obese adult patients | Attitudes toward obese adult patients | From all departments and administrative levels of the hospital, working full-time or part-time | A nonexperimental, descriptive, correlational study | BATOS | Favourable attitudes toward obese adults among 81% of nurses. More favourable attitudes among nurses with a higher BMI. No significant correlation between the nurse’s level of education and the nurse’s attitude score. Sensitivity training: ranked as the most influential factor that would positively improve their attitude. |
| 33 | Llewellyn et al. (2023) | JA | USA | Pre-survey: 47 (85%F), post-survey: 73 (80%F), first semester pre-license baccalaureate nursing students | To identify differences in results on the BAOP and positive/negative adjective attribution – To identify student perceptions of patient weight and the simulation in their narrative answers | Weight bias | _ | Pre-test post-test mixed-methods parallel convergent design QUANT + QUAL | Intervention (LEARN model and a simulation scenario) - FPS – BAOP – Qualitative questions | Significantly lower BAOP scores for agreeing that obesity results from: lack of love or attention, overeating, not exercising, eating more than non-obese people, or poor eating habits. Positive trends for 5 adjectives (will power, self-control, endurance, strength, and shapeliness), and a trend from overeating to neutral. Pre-survey themes: no different treatment for obese people, educating them about impacts of obesity. Post-survey themes: providing care based on patient-centred priorities, listening, making adaptions. |
| 34 | Marcum (2009) | D | USA | 101 (100F) participants (26 RNs, 21 nursing instructors, 31 undergraduate nursing students, 20 graduate nursing students, and 3 other) | To determine whether obesity sensitivity education was an effective intervention in changing nurses’ attitudes and beliefs about obese people | Attitudes and beliefs about obese people | 19 participants working in a designated obesity facility | Quasi-experimental mixed-method study (pre-test/post-test) | Obesity Sensitivity Education (intervention) - ATOP – BAOP – An open-ended question (for additional revelation of attitudes and beliefs) | No change in attitudes toward obese people. Improvement in beliefs about obese people among graduate nursing students, obese respondents, and those employed at a designated obesity facility. Answers to the open-ended question: a sense of awareness toward obese patients and a decrease in judgmental remarks. |
| 35 | Maroney & Golub (1992) | JA | USA | 67 nurses (65F) | To assess the attitudes of US nurses toward obese people – To compare those attitudes with those held by the Canadian nurses – To explore whether those nurses who have negative attitudes toward obese patients would also have ethnic prejudices | Attitudes toward obese people – Ethnic prejudices | Community hospital - Private nursing home | Quantitative | A questionnaire of 20 questions about attitudes toward obese persons, plus four questions regarding ethnic attitudes | Stronger weight controllability belief, repulsion at caring for an obese patient, and less empathy among Canadian nurses. Weight controllability beliefs and belief of the necessity to be put on a diet while in hospital, among the majority of nurses. Perception of care as exhausting and stressful, and the preference to avoid it if given a choice. Stronger perception of obese Caucasian adults as aggressive, compared to the general obese population. A significant positive correlation between attitudes toward obesity and ethnic prejudices. |
| 36 | Molloy et al. (2016) | JA | USA | 70 first-semester nursing students (93%F) | To create an effective intervention for helping nursing students to self-identify their current attitudes and beliefs about caring for obese individuals | Attitudes and beliefs about caring for obese individuals | _ | A 1-group repeated-measures design (surveys immediately before the intervention, immediately after the intervention, and 30 days after the intervention) | BSI – NATOOPS - BAOP | Improvement in student attitudes toward obesity and obese patients (decreased NATOOPS scores, in both immediate and 30-day post-intervention). Significant improvement in student beliefs (increased BAOP scores, in both immediate and 30-day post-intervention). |
| 37 | Moyo (2022) | MT | Namibia | 250 nurses (162F) | To measure the levels of knowledge, attitude, and practices of nurses – To determine if there was an association between nurses’ knowledge, attitude, and practice scores | Attitudes and practices regarding obesity | Private healthcare institutions | A quantitative cross-sectional design | A self-administered questionnaire | Good attitudes and good practices regarding obesity among 44.8% and 38.4% of participants, respectively. Statistically significant strong positive correlation between knowledge and attitude scores, knowledge and practice scores, and attitude and practice scores. |
| 38 | Mullaney (2016) | HT | USA | 7 nursing students (4F) | Quantitative: to determine whether certain participant characteristics may suggest more or less negative attitudes towards obese persons – Qualitative: to determine what each person learned during the presentation and whether they believe it changed their perspectives on people affected by obesity | Weight stigma | _ | A mixed-methods prospective design (a cross-sectional post-test only) | Health Education Presentation Intervention – Questionnaire including questions to determine attitudes towards obesity or beliefs about obese persons after they witnessed the presentation, and questions regarding the impact of the intervention | Very low levels of externally reported bias. Slightly more anti-fat attitudes among females (especially regarding physical care provision). Nearly all participants were surprised with the information presented regarding barriers to weight loss or with prevalence of stigma in healthcare. |
| 39 | Nicholls (2016) | JA | UK | 92 student nurses (85F, 3M, 4 not identified) | To examine whether the level of advice offered to obese patients by student nurses is associated with (i) the perceived causal factors of obesity, and (ii) attitudes towards obesity | Perceived causal factors of obesity - Attitudes towards obesity | _ | 1^st^ aim: an experimental design (a between subjects design) – 2^nd^ aim: a quasi-experimental design | Four vignettes (for causes of obesity) – FPS short form | No association between the level of advice offered and either the causal factor of obesity, or the student nurses' attitude towards obesity. |
| 40 | Nickel et al. (2019) | JA | Germany | 949 participants, including 150 nurses (109F) & 202 nurses in training (166F) | To evaluate the stigmatization of obesity | Weight bias | _ | A randomized study | FPS | Lowest FPS among nurses and the highest among medical students (from subgroups of the intervention group). |
| 41 | Obitz & Frensborg (2014) | BT | Costa Rica | 7 RNs (4F) | To explore if nurses view and experience the healthcare environment as a stigmatizing place for obese patients – To explore if they experienced any negative attitudes that may affect the care of obese patients | Nurses’ attitudes and experiences of caring for patients with obesity | Different areas at the hospitals, emergency room and surgery | Qualitative study with semi-structured interviews | Interviews in four different areas (perception of obesity and causes of obesity; perception of the obese patients’ feelings; attitudes in the healthcare environment with obesity; and needs of improvement in the healthcare environment) | Five identified themes: experiences of inadequate resources (material and personnel) in the care of obese patients, importance of maintaining a caring relationship with an obese patient, attitudes as a consequence of the view of causes of obesity (uncontrollable or self-inflicted), experiences of stigmatization of obese patients (in healthcare; but they thought they had not contributed to it), and nurses' perspectives on improvements in the healthcare environment (importance of patient and nurse education). No particular difference between attitudes of male and female participants. |
| 42 | Oliver et al. (2020) | JA | USA | 17 clinical groups, each including six to eight students: 125 third-year nursing students (119F) | To determine whether negative attitudes and beliefs toward those with obesity can be improved | Attitudes and beliefs toward those with obesity | _ | A one-group pre-test post-test repeated measures, concurrent mixed-methods study | CeWebs training – ATOP - BAOP | Increased ATOP and BAOP scores, in the post-training stage, indicating more positive attitudes and less weight controllability beliefs. |
| 43 | Oliver et al. (2021a) | JA | USA | 197 third‐year baccalaureate nursing students (187F) | To report nursing students' perspectives of perceived and observed weight bias by HCPs practicing in the healthcare setting, as reported through reflective journal entries | Observed weight stigma | _ | A descriptive qualitative study design, involving reflective journaling and qualitative content analysis | Self‐reflective journals (responding to five open‐ended questions) | Three themes: 1) Direct impact: Observed implicit and explicit provider weight bias, 2) Indirect impact: Weight bias due to skills, equipment, staffing, or environmental deficits, 3) Reactions toward HCPs’ weight bias: Conflict between weight bias training and real‐world healthcare experiences |
| 44 | Oliver et al. (2021b) | JA | USA | 98 third-year baccalaureate nursing students (94F), enrolled in a medical-surgical clinical practicum – 280 journal entries | To explore the use of reflective journals as a tool to raise self-awareness and self-reflection of weight bias as part of a weight sensitivity training program | Self-awareness and self-reflection of weight bias | _ | Qualitative study | Completing five journal entries | Four themes: 1) higher self-awareness of personal weight bias; 2) recognition of obesity as a chronic disease and not a choice; 3) insufficient proper resources or training may perpetuate weight bias; 4) opportunity for weight sensitivity training to improve patient care. |
| 45 | Oliver et al. (2022) | JA | USA | 99 baccalaureate nursing students (92F, 6M, 1 other), in the first semester of the third year of their nursing curriculum (13 medical-surgical clinical practicum groups, each consisting of 6-8 students) | To explore whether case-based learning will enhance a WBR programme among nursing students to improve their attitudes and beliefs towards persons with obesity | Weight bias | _ | Two-arm, CRT | WBR programme modules (standard or case-based) – ATOP - BAOP | Significantly improved BAOP score among the group that received case-based WBR, compared to controls (standard WBR), indicating improved beliefs about the controllability of obesity. No significant changes to attitudes about individuals with obesity (ATOP scores). |
| 46 | Oliver et al. (2023) | JA | USA | 7 NP students (6F, 1 other) | To assess the effect of a WBR intervention that included SBE with SPs living with obesity and an educational session on attitudes and beliefs toward persons with obesity | Weight bias | _ | Pilot study | WBR intervention – ATOP - BAOP | No statistically significant differences in pre-intervention and post-intervention score regarding ATOP and BAOP questionnaires. |
| 47 | Oliver et al. (2024) | JA | USA | 18 NP students (17F), during their final clinical year | To explore the efficacy of a WBR intervention, including SBEs with SPs living with obesity, in reducing weight bias | Weight bias | _ | A one-group, repeated measures study | ATOP – BAOP – NATOOPS – 3 SBEs, with each session accompanied by an interactive WBR intervention | Improvement in all weight bias measure, however, not statistically significant. |
| 48 | Ozaydin & Tuncbeden (2022) | JA | Turkey | 233 students (205F) in their second, third, and fourth years in the faculty of nursing | To determine the prejudice and stigmatization levels of nursing students towards obese individuals | Weight stigma – Stigmatization tendency | _ | Descriptive and correlational study | GAMS-27 obesity prejudice scale - Stigma scale | Significantly higher level of prejudice among senior students, compared to the third-year students, and among students with extended families compared to the ones with nuclear families. A positive, weak, and significant relationship between the obesity prejudice and the level of stigmatization. No relationship between obesity prejudice and BMI. |
| 49 | Petrich (2000) | JA | USA | 102 third- and/or fourth-year nursing students, in addition to 28 medical students | To compare perceptions of obesity among medical and nursing students | Perceptions of obesity | _ | Qualitative descriptive study | A questionnaire modified by researcher based on the literature (6 open-ended questions for students to describe their perceptions of obesity) | Similarities: most prevalent theme: feeling repulsed at the appearance of obese patients (only male patients) – perception of obese people as unhealthy, inactive, lazy, and lacking self-control. Differences: 29% more medical students felt disgusted at the appearance of obese patients, 19% more perceived an obese person as inactive and lazy. 14% more nursing students did not like the terms “fat,” “overweight,” and “obese”, while perceiving the terms as negative labels, and more nursing student felt empathy toward obese women. |
| 50 | Pfeiffer (2017) | D | USA | 256 participants (235F), including 37.9% RNs, 3.2% NPs, 4.7% CNSs, 0.8% LPNs | To explore the impact of patients’ weight on nurses’ attitudes and care decisions | Attitudes toward obese patients – Care decisions | 20 different medical specialty areas - Most common ones: emergency or critical care, medical-surgical, nursing management, outpatient/ambulatory care | A 2x2x2 factorial experiment, with randomized assignment | Anonymous online survey, including: patient photographs, clinical vignettes and care decisions, 14 selected items from ATOP and several developed new items | Far more positive (than negative) attitudes toward patients of both weights among nurses, despite a significant negative influence of patients’ weight upon nurses’ attitudes. No significant impact of patients’ weight on nurses’ care decisions. Neither the patient’s weight nor the nurse’s attitudes toward the patient influenced anticipated number of walk assists. |
| 51 | Poon & Tarrant (2009) | JA | Hong Kong | 352 undergraduate student nurses (308F, 44M) & 199 RNs (169F, 30M) | To investigate undergraduate student nurses’ and RNs’ attitudes towards obese persons and towards the management of obese patients | Attitudes towards obese persons and the management of obese patients | RNs: medical/surgical (n=36) – obstetrics/gynaecology (4) – critical care (5) – emergency (10) – psychiatry (20) – paediatrics (7) – geriatrics (10) – community health (38) – outpatient clinic (25) – other (40) | Descriptive cross-sectional study | FPS – ATOAP | Average fat-phobia and neutral attitudes towards management of obese patients. Significantly higher levels of fat-phobia and more negative attitudes among RNs. Perceptions of the majority from obese people: they liked food, were more likely to overeat and were shapeless, slow, and unattractive. Almost half of participants believed obese people should be put on a diet while in hospital, and that caring for them is physically exhausting. |
| 52 | Robstad et al. (2018a) | JA | Norway | 30 qualified intensive care nurses (24F) | To design and test research instruments to measure qualified intensive care nurses’ implicit and explicit attitudes and behavioural intentions toward obese intensive care patients | Implicit and explicit attitudes and behavioural intentions toward obese patients | A general ICU | A cross-sectional pilot study | IATs (for both implicit attitudes and stereotypes) – AFA - Explicit bias scales (for feelings and beliefs about fat and thin people) - Vignettes describing workplace scenarios (for behavioural intentions) | Implicit and explicit preferences for thin over thick people. Perceiving obese patients lazier than normal-weight ones. Intention of nurses to help obese patients immediately. Negative correlation between such intentions for assistance and explicit anti-fat attitudes and implicit anti-obese stereotypes. Reliability of behavioural intentions and anti-fat attitudes. Satisfactory face validity, convergent validity, and discriminant validity, both within and between implicit and explicit attitudes and stereotypes. |
| 53 | Robstad et al. (2018b) | JA | Norway | 13 qualified ICU nurses (13F) | To obtain a deeper understanding of qualified intensive care nurses’ experiences of caring for obese patients in intensive care | Experiences of caring for obese patients in intensive care | ICUs at two different hospitals | A qualitative hermeneutic approach | Semi-structured individual interviews | Ambivalent feelings while caring for obese patients by nurses: while they endeavoured to provide good and equal care to all patients, they simultaneously held negative beliefs and attitudes towards obese patients, resulting in a picture of the obese patient as being different than other patients. Nurses perceiving caring for obese patients as emotionally demanding because of their attitudes and beliefs about these patients. Nurses having a desire to improve their attitudes toward obese patients. |
| 54 | Robstad et al. (2019) | JA | Norway | 159 qualified ICU nurses (134F) | To examine qualified ICU nurses’ implicit and explicit attitudes towards obese ICU patients – To examine if their attitudes are associated with their behavioural intentions towards these patients | Implicit and explicit attitudes and behavioural intentions toward obese patients | General, medical, or surgical ICUs | A cross‐sectional web‐based study | IATs (for both implicit attitudes and stereotypes) - Four explicit bias scales measuring feelings and beliefs about thin and thick people – AFA - Vignettes measuring behavioural intentions | Significant overall implicit preference for thin over thick people. No significant between‐group differences in implicit and explicit attitudes related to self‐reported weight stature. Higher scores on the AFA willpower subscale among males. Neither implicit nor explicit attitudes and stereotypes were associated with behavioural intention. Less intention among males to help obese patients immediately. Perceiving obese patients slightly ‘worse’ and ‘lazy’, comprising less willpower than thin individuals |
| 55 | Rodriguez-Gazquez et al. (2020) | JA | Spain | 578 undergraduate nursing students (467F), from all four academic years | To analyse if the anti-fat attitudes of nursing students change during their degree training | Anti-fat attitudes | _ | Cross-sectional study | AFA questionnaire, including three domains: fear of fat, willpower, dislike | Highest AFA score among 1^st^ year students, and lowest ones among last year students. Decrease in anti-obesity attitudes as students progressed in their degree (despite the slight increase from the 2^nd^ to the 3^rd^ year). Lowest dislike and willpower scores among females. |
| 56 | Salziyan (2018) | JA | Malaysia | 297 nursing students in a private college (267F) | To determine the correlation between BMI of nursing students and their attitude towards obesity | Attitude towards obesity | _ | A cross-sectional study | ATOP | More favourable (rather than negative) attitudes toward obese people, among participants. No significant correlation between nursing students’ BMI and their attitude scores. |
| 57 | Shea & Gagnon (2015) | JA | Canada | 11 ICU nurses (9F), having at least 1 previous experience of providing care to a PLWO in the ICU | To examine the experiences of ICU nurses who work with PLWO using the Othering framework developed by Canales in 2010 | Experiences of working with PLWO in ICU | 2 ICUs | A qualitative design, incorporating explorative and interpretive properties | Interviews | Four themes, including ‘Exclusionary Othering in the ICU’, with this subtheme: ‘Witnessing instances of obesity bias’. Other than physical challenges, nurses were challenged by their own past experiences as well as their experiences with colleagues who displayed negative and discriminatory attitudes toward PLWO. |
| 58 | Sikorski et al. (2013) | JA | Germany | 682 HCPs, including 321 nurses | To investigate HCPs’ attitudes towards overweight and obesity | Attitudes towards overweight and obesity | _ | Quantitative | Questionnaire on stigmatizing attitudes (Short form of FPS based on a vignette describing a female obese patient) and perceived causes of obesity | Slightly more positive attitudes among nurses, compared to physicians, therapists, and other medical staff. Perception of obesity as an illness to a greater extent among nurses (biomedical causes and less stigmatizing), while physicians attributed obesity to the individual. |
| 59 | Snethen et al. (2014) | JA | USA | 332 undergraduate nursing students; including 158 pre-nursing students and 174 nursing students enrolled in the clinical nursing major | To examine the attitudes and beliefs of undergraduate pre-nursing students and students in the clinical nursing major about children who are overweight or obese – To examine whether there are differences or similarities in those attitudes and beliefs between the two groups of students | Attitudes and beliefs about children who are overweight or obese | _ | A descriptive study, using a cross-sectional design | Attitudes Toward Obese Persons Scale: Children’s Version (ATOP & BAOP, modified for children, and combined) | Negative perceptions of overweight children among both groups. More prevalent reports among pre-nursing students about overweight children: untidy, having different personalities, resenting normal-weight children, more emotional than non-overweight children, should not expect to lead normal lives, having poor eating habits that lead to their overweight. Higher chance of students in the clinical nursing major to report ‘being sociable’ about overweight children. |
| 60 | Styk et al. (2024) | JA | Poland | 119 Nigerian 1^st^ year nursing students (86F) & 120 Polish 1^st^ year nursing students (81F) | To examine and compare weight bias among nursing students from two separate cultures – To adapt and translate FPS into Polish | Attitudes and beliefs towards people with obesity – Fatphobia | _ | Cross-cultural | ATOP – BAOP - FPS | Significantly higher ATOP and BAOP score among Polish students (showing more positive attitudes and beliefs), compared to the Nigerian ones. No significant difference between the two cultural groups in terms of FPS scores. Significantly higher ATOP scores among Polish females, compared to their male counterparts; as well as among Nigerian males, compared to their female counterparts. Negative correlation between BMI and FPS among Nigerian students, as well as between BMI and ATOP among Polish ones. No correlation between age and weight bias scores. |
| 61 | Swift et al. (2013) | JA | UK | 1130 trainee HCPs, including Nursing MNurSci and Nursing BSc students (n=NM) | To determine the extent of weight bias among UK trainee HCPs | Weight bias | _ | Cross-sectional survey | FPS - BAOP | Significantly higher chance of Nursing BSc students to have had ‘a lot’ or ‘some’ contact with obese people, compared to Medicine and Nutrition students. Beliefs of lower weight controllability among Nursing BSc students compared to Dietetics, Medicine and Nursing MNursSci students. Lower fat-phobia in Nursing BSc students, compared to all other groups. |
| 62 | Tanneberger & Ciupitu-Plath (2018) | JA | Germany | 73 nurses (78.3%F) | To explore whether nurses’ weight controllability beliefs influence their perception of how care is provided to obese patients | Weight controllability beliefs – Perception of provision of care | An acute care clinic, providing both basic and specialized inpatient health care services | A cross-sectional, exploratory, quantitative study design | WCB subscale of AFAT – Reports on perception of discrimination in, and available resources for, the provision of care to obese patients | Significant associations between nurses’ weight controllability beliefs and their perception of obese patients being treated differently compared to other patients both by other nurses, and by themselves. |
| 63 | Tanner (2017) | D | USA | G1 (4F, 0M), G2 (40F, 1M),  traditional baccalaureate nursing students in their junior year of study | To determine the effect of an educational intervention about weight bias in HCPs on the attitudes of undergraduate nursing students | Weight bias | _ | Quasi-experimental study using pre-test post-test design | Yale Rudd Centre Weight Bias in Healthcare Video (intervention) - FPS | Low levels of fat-phobia at baseline. Statistically significant increase in fat-phobia levels after the intervention (still in the positive/neutral range of fat-phobia). Being “undisciplined” and “unappealing”: strongest negative stereotypes about overweight people. |
| 64 | Thompson et al (2021) | JA | USA | Paediatric surgery nurses (n=108, 97.2%F) & school nurses (n=177, 98.9%F) | To describe paediatric surgery nurses' and school nurses' weight bias towards children with overweight or obesity | Weight bias towards children with overweight or obesity | _ | Observational and cross-sectional study | The Attitudes of Health Care Providers about Treating Patients with Obesity scale | Agreement among nurses about the importance of treating patients with obesity with compassion and respect. Negative attitudes towards patients with obesity (perceiving them as non-compliant and hard to deal with). Commonly hearing/witnessing negative comments or stereotypes about patients with obesity from other professionals in their fields. Association between the percentage of time spent working with overweight children and more positive weight attitudes. |
| 65 | Turner (2024) | JA | USA | 177 paediatric HCPs, including 136 RNs and LPNs | To compare implicit and explicit weight bias among nurses and other HCPs | Implicit and explicit weight bias | _ | Cross-sectional design | IAT - AFA | Significantly higher implicit weight bias among nurses, compared to physicians and advanced practice providers. No significant difference between nurses and other HCPs in terms of explicit weight bias. |
| 66 | Tüzün et al. (2023) | JA | Turkey | 495 HCPs, including 180 nurses (98.8%F) | To explore if beliefs and negative attitudes towards obese patients among HCPs differ based on profession | Attitudes and beliefs towards obese patient | _ | Cross-sectional study | ATOP scale and BAOP scale | Significantly more favourable beliefs about obese patients among nurses, compared to physicians. No significant difference in different groups of HCPs, regarding negative attitudes. |
| 67 | Usta et al. (2021) | JA | Turkey | 685 nursing students (525F) | To determine the beliefs, attitudes, and phobias of nursing students about obese individuals | Beliefs, attitudes, and phobias about obese individuals | _ | Descriptive and cross‐sectional design | FPS – ATOP (Turkish version) – BAOP (Turkish version) | Moderate levels of fat-phobia and attitudes towards obese individuals, and weight controllability beliefs among nurses. Higher fat-phobia and stronger weight controllability beliefs among females. Lower fat-phobia among obese participants. Higher fat-phobia among the calorie counting participants. More positive attitude among 4^th^ compared to 1^st^ year students. Less positive attitudes and stronger weight controllability beliefs among the ones with a dieting history. No relationship between the eagerness to give care to patients with obesity and fat-phobia and beliefs. More negative attitudes among those who were unwilling to give care. |
| 68 | Van der Voorn et al. (2023) | JA | The Netherlands | 555 HCPs treating children and adolescents with obesity, including 223 youth healthcare nurses | To study the interdisciplinary differences of weight-biased attitudes of Dutch HCPs | Weight bias | _ | Cross-sectional study | Attitudes of Health Care Providers about Treating Patients with Obesity scale | Significant higher perceived frustrations among youth healthcare physicians, compared to nurses. Significant higher perceptions of weight bias by colleagues reported by GPs, compared to nurses. |
| 69 | Waller et al. (2012) | JA | USA | 45 nursing students (86.67%F), in addition to 45 psychology students | To determine the implicit or unconscious attitudes of participants towards overweight individuals | Implicit weight bias | _ | A mixed design experiment with one between-subjects variable (student major) and one within-subjects variable (congruent or incongruent) | Computerized IAT | Presence of a statistically significant implicit bias towards overweight individuals among nurses. No significant difference between nursing and psychology students regarding implicit weight bias. |
| 70 | Wang et al. (2016) | JA | China | 297 RNs (all women) | To examine attitudes toward obese patients among nurses – To explore the relationship between weight bias and nurses’ weight locus of control | Attitudes toward obese patients | Medical, emergency, and surgical units, in community health service centres | Cross-sectional survey | ATOP – eWLOC | Slightly positive attitudes toward obese persons. A tendency to think that obesity was beyond the control of one’s ability. Marginally positive correlation between BMI and favourable attitudes toward obese persons. Significantly positive correlation between external locus of control and favourable attitudes toward obese persons. More positive attitudes among RNs in specialist departments, compared to peers in medical and surgical ones. |
| 71 | Ward-Smith & Peterson (2016) | JA | USA | 358 NPs (94%F) | To describe the self-reported attitudes and beliefs of NPs toward overweight and obese individuals | Attitudes and beliefs toward overweight and obese individuals | _ | Descriptive study | ABNPS | NPs perceiving obese individuals as inferior compared to others, in terms of being good, success, marriage suitability, tidiness, health, and sociability. Viewing obesity as a result of overeating, lack of physical activity, or a biological disorder. |
| 72 | Watson et al. (2008) | JA | Canada | 626 RNs (598F) | To develop and test an instrument to measure nurses’ attitudes towards obesity and obese adult patients | Attitudes towards obesity and obese adult patients | Medical surgical care - community outreach and education - critical care – Emergency care | Methodological study | NATOOPS | 36 items, five factors: 1) Response to obese patients, 2) Characteristics of obese individuals, 3) Controllable factors contributing to obesity, 4) Stereotypic characteristics of obese patients, 5) Supportive roles in caring for obese patients. Cronbach’s alpha=0.81 for the reduced scale, with a range of 0.45 to 0.79 on the five factors. Appropriate construct validity (significant differences between contrast groups on three factors and consistency with underlying theory). |
| 73 | Williams-Hailey (2015) | D | USA | Phase 1: 6 expert panellists (experienced psychiatric nurses) – Phase 2: 149 psychiatric RNs (131F) | 1)To develop an instrument to measure the knowledge, attitudes, and self-reported behaviour of psychiatric nurses towards the mentally ill obese patient – 2)To determine if psychiatric nurses’ knowledge, attitudes and behaviours are different when the patient is obese versus normal weight | Knowledge, attitudes, and self-reported behaviours towards the mentally ill obese patient | Working with adult psychiatric patients | Quantitative study using a descriptive comparative design | Phase 1: four subsections of the instrument: knowledge, general attitudes towards obesity, intrinsic attitudes, and the self-reported behaviours – Phase 2: the instrument in phase 1, two developed vignette scenarios | No obtained reliability for the instrument as a whole. More biased responses from the obese patient vignette (on these items: “strong-willed/weak-willed, sociable/not sociable, attractive/unattractive, trusting/suspicious”). More bias among the older nurses towards the obese patient. Bias towards the obese patient in the vignette regarding self-reported behaviours. |
| 74 | Wise et al. (2014) | JA | Australia | 221 rehabilitation HCPs, including 70 nurses | To compare nurses and physiotherapists in terms of their beliefs and attitudes to obesity | Beliefs and attitudes to obesity | Public and private rehabilitation facilities | A cross-sectional, anonymous, self-report survey | FPS – BAOP | Significantly lower levels of fat-phobia among nurses, compared to physiotherapists (Higher number of younger people among physiotherapists, so after matching age and sex, the difference became non-significant). |
| 75 | Wynn et al. (2018) | JA | UK | 372 HCPs, including 61 nurses (52F) | To investigate differences in obesity prejudice amongst HCPs | Obesity prejudice | _ | Quantitative | ATOP | Nurses’ mean ATOP was more than that of operating department practitioners, pharmacists, junior doctors, and consultants, and less than that of dietitians, health care assistants, and medical students. Whether these differences were statistically significant or not, was not mentioned. |
| 76 | Yılmaz & Yabancı Ayhan (2018) | JA | Turkey | 437 nursing students | To investigate nursing students’ attitudes and behaviours towards obese individuals | Attitudes and behaviours towards obese individuals | _ | A descriptive and sectional study | FPS - ATOP | Moderate and high level of negative attitudes towards overweight and obese individuals among student nurses. |
| 77 | Yılmaz & Yabancı Ayhan (2019) | JA | Turkey | 190 student nurses (161F) & 189 RNs (183F) | To assess whether there is prejudice toward obese persons among student nurses and RNs | Weight bias | _ | Quantitative | FPS – BAOP | Average levels of fat-phobia for both groups. Presence of negative beliefs about obese persons among the majority of both groups. More negative prejudices and negative beliefs among RNs, compared to student nurses. More positive attitudes among obese participants and the ones having an obese family member. |
| 78 | Young (1985) | MT | USA | 59 RNs (59F) | To determine if there is a difference in the attitudes toward obesity between obese and non-obese nurses, and a relationship between the attitudes toward obesity and the personal or professional characteristics of the nurses, or nurses' previous experiences with obesity | Attitudes toward obesity | 6 medical (general medicine units, neurology, endocrine-renal medicine) & 6 surgical units (general surgical units, urology, otolaryngology-eye, orthopaedic, neurosurgery-plastics) | A correlational descriptive survey | The Obesity Questionnaire | A median attitude score (neither extremely negative nor positive). No statistically significant differences in attitudes toward obesity between obese and non-obese subjects. No statistically significant relationship between the attitudes toward obesity and nurses’ previous experiences with obesity/ personal characteristics/ professional characteristics. A positive statistically significant relationship between proportion of obese patients in their daily care and positive attitudes. |
| 79 | Zhu et al. (2013) | JA | UK | 399 RNs (354F) | T examine whether attitudes towards obese people would directly influence weight management practices | Attitudes towards obese people | _ | Cross-sectional study | ATOP | Neutral attitudes towards obese people among RNs. No significant effect of nurses’ attitudes towards obese patients on their self-efficacy and weight management practices. |
| 80 | Zuzelo & Seminara (2006) | JA | USA | 119 full-time RNs | To describe RNs’ attitudes toward obese adult patients – To explore the relationship between RNs’ attitudes toward obese adult patients, and education, body weight, years of experience, and work setting – To explore the difference in attitudes toward obese adult patients between RNs working in hospital, acute rehabilitation, or skilled care facilities | Attitudes toward obese patients | Medical centre – Acute rehabilitation institution – Skilled nursing facility | Non-experimental descriptive study | NATOOPS – A qualitative question to measure RNs’ practices and reactions to obese adults | No significant relationship between attitude scores and demographic variables (body weight, years of RN experience, education level, and type of work setting). Positive attitudes of RNs toward obese adults. Higher NATOOPS score among medical centre RNs, compared to acute rehabilitation ones. Qualitative themes: believing obese patients deserve equal treatment – recognizing unique care needs – feeling overwhelmed by care needs – effort to avoid hurtful encounters – feeling astounded – feeling sympathy – dreading the physical care demands – worrying about personal safety |

Abbreviations: ABNPS: Attitudes and Beliefs of NPs Survey – AFA: The Anti-fat Attitudes – AFAT: The Anti-fat Attitudes Test – ANTOAP: Attitudes of Nurses Toward Obese Adult Patients – ATOAP: Attitudes Toward Obese Adult Patients – ATOP: Attitudes Toward Obese Persons – ATOPS: The attitudes toward obese persons scale – BAOP: Beliefs About Obese Persons – BATOS: The Bray Attitude Toward Obesity Scale – BMI: body mass index – BSI: Bariatric Sensitivity Intervention – BSN: bachelor of science in nursing – BT: Bachelor’s thesis – CAO: Causal Attributions for Obesity – CNA: certification in nursing assistance – CRT: cluster-randomized controlled trial – D: dissertation – eWLOC: External Weight Locus of Control Subscale – F: female – FACT: Full-time Accelerated Coursework Track - FPS: The Fat Phobia scale – G: group - HAES: health at every size – HCP: health care professional – HT: honours thesis – IAT: implicit association test – ICU: intensive care unit – JA: Journal article - JSE-HPS: Jefferson Scale of Empathy-Health Professions Students – JSENS: Jefferson Scale of Empathy for Nursing Students - LKM: loving-kindness meditation – LPN: license practical nursing – M: male – MBET-OSS: Mindfulness-based empathy training and obese simulation suits - MT: Master’s thesis – NATOOPS: Nurses’ Attitudes Towards Obesity and Obese Patients Scale – NB: non-binary – NM: not mentioned – NP: nurse practitioner – Nursing BSc: Bachelor of Science in Nursing – Nursing MNurSci: Master of Nursing Science – OPS: Obesity perception survey – PLWO: patient living with obesity – Qual: qualitative – Quant: quantitative – RCT: Randomized Control Trial – RN: registered nurse – RNA: Registered Nurse Anaesthetists – SBE: Simulation-based experiences – SP: Standardised Participant - SSI: Stigmatizing Situation Inventory – TG: transgender – WBIS-M: Modified Weight Bias Internalized Scale – WBR: weight bias reduction – WCB: Weight Control/Blame

**Appendix 4: Descriptors represented by each included study**

| Authors (year) | Descriptors | | | | | | | |
| --- | --- | --- | --- | --- | --- | --- | --- | --- |
|  | Description | Exploring associations | Comparing different groups of nurses | Comparing nurses with other health professionals | Intervention assessment | Finding solutions | Exploring consequences or causes | Instrument development and psychometrics |
| Akman et al. (2010) |  |  |  | ✓ |  |  |  |  |
| Alexander (2018) |  | ✓ |  |  |  |  |  |  |
| Bagley (1989) | ✓ | ✓ | ✓ |  |  |  |  | ✓ |
| Barra & Singh Hernandez (2018) | ✓ |  |  |  | ✓ |  |  |  |
| Bottcher & Chao (2022) |  | ✓ |  |  |  |  |  |  |
| Brown & Thompson (2007) | ✓ |  |  |  |  |  |  |  |
| Bucher Della Torre et al. (2018) |  |  |  | ✓ |  |  |  |  |
| Can Gür & Yılmaz (2024) |  |  |  |  | ✓ |  |  |  |
| Culbertson & Smolen (1999) | ✓ | ✓ | ✓ |  |  |  |  |  |
| Darling & Atav (2019) |  |  | ✓ | ✓ |  |  |  |  |
| Dunagan et al. (2016) | ✓ |  |  |  |  |  |  |  |
| Dunham (2024) | ✓ | ✓ |  |  | ✓ |  |  |  |
| Frick (2007) |  |  |  | ✓ |  |  |  |  |
| Fruh et al. (2019) |  |  |  |  | ✓ |  |  |  |
| Gajewski (2023) |  |  |  |  | ✓ |  |  |  |
| Gamaly (2022) |  |  |  |  | ✓ |  |  |  |
| Garcia (2012) |  |  | ✓ |  |  |  | ✓ |  |
| Garner & Nicol (1998) | ✓ |  | ✓ |  |  |  |  |  |
| Geckle (2001) | ✓ |  | ✓ |  |  |  |  |  |
| George et al. (2019) | ✓ | ✓ |  |  |  |  |  |  |
| Gormley & Melby (2020) | ✓ |  | ✓ |  |  |  |  |  |
| Gujral et al. (2011) |  | ✓ |  |  | ✓ |  |  |  |
| Halvorson et al. (2019) | ✓ |  |  | ✓ |  |  |  |  |
| Härgestam et al. (2024) | ✓ |  |  |  |  |  | ✓ |  |
| Hartman (2017) |  | ✓ | ✓ |  |  |  |  |  |
| Hauff et al. (2019) | ✓ |  |  |  |  | ✓ |  |  |
| Hauff et al. (2020) | ✓ |  | ✓ |  |  |  |  |  |
| Hemati & Zokaei (2016) |  |  | ✓ |  |  |  |  |  |
| Johnson (2018) | ✓ |  | ✓ |  |  |  |  |  |
| Joseph (2022) |  | ✓ |  |  | ✓ |  |  |  |
| Kerbyson (2023) | ✓ |  |  |  |  |  | ✓ |  |
| Lilliott (2000) | ✓ |  | ✓ |  |  | ✓ |  |  |
| Llewellyn et al. (2023) |  |  |  |  | ✓ |  |  |  |
| Marcum (2009) |  |  |  |  | ✓ |  |  |  |
| Maroney & Golub (1992) | ✓ | ✓ | ✓ |  |  |  |  |  |
| Molloy et al. (2016) |  |  |  |  | ✓ |  |  |  |
| Moyo (2022) | ✓ | ✓ |  |  |  |  |  |  |
| Mullaney (2016) | ✓ |  | ✓ |  | ✓ |  |  |  |
| Nicholls (2016) |  | ✓ |  |  |  |  |  |  |
| Nickel et al. (2019) |  |  |  | ✓ |  |  |  |  |
| Obitz & Frensborg (2014) | ✓ |  | ✓ |  |  | ✓ | ✓ |  |
| Oliver et al. (2020) |  |  |  |  | ✓ |  |  |  |
| Oliver et al. (2021a) | ✓ |  |  |  |  |  |  |  |
| Oliver et al. (2021b) |  |  |  |  | ✓ |  |  |  |
| Oliver et al. (2022) |  |  |  |  | ✓ |  |  |  |
| Oliver et al. (2023) |  |  |  |  | ✓ |  |  |  |
| Oliver et al. (2024) |  |  |  |  | ✓ |  |  |  |
| Ozaydin & Tuncbeden (2022) |  | ✓ | ✓ |  |  |  |  |  |
| Petrich (2000) |  |  |  | ✓ |  |  |  |  |
| Pfeiffer (2017) | ✓ |  |  |  |  |  | ✓ |  |
| Poon & Tarrant (2009) | ✓ |  | ✓ |  |  |  |  |  |
| Robstad et al. (2018a) | ✓ | ✓ |  |  |  |  |  | ✓ |
| Robstad et al. (2018b) | ✓ |  |  |  |  |  |  |  |
| Robstad et al. (2019) | ✓ | ✓ | ✓ |  |  |  |  |  |
| Rodriguez-Gazquez et al. (2020) | ✓ |  | ✓ |  |  |  |  |  |
| Salziyan (2018) | ✓ | ✓ |  |  |  |  |  |  |
| Shea & Gagnon (2015) | ✓ |  |  |  |  |  |  |  |
| Sikorski et al. (2013) |  |  |  | ✓ |  |  |  |  |
| Snethen et al. (2014) | ✓ |  | ✓ |  |  |  |  |  |
| Styk et al. (2024) |  | ✓ | ✓ |  |  |  |  | ✓ |
| Swift et al. (2013) |  |  |  | ✓ |  |  |  |  |
| Tanneberger & Ciupitu-Plath (2018) |  | ✓ |  |  |  |  |  |  |
| Tanner (2017) | ✓ |  |  |  | ✓ |  |  |  |
| Thompson et al (2021) | ✓ | ✓ |  |  |  |  |  |  |
| Turner (2024) |  |  |  | ✓ |  |  |  |  |
| Tüzün et al. (2023) |  |  |  | ✓ |  |  |  |  |
| Usta et al. (2021) | ✓ | ✓ | ✓ |  |  |  |  |  |
| Van der Voorn et al. (2023) |  |  |  | ✓ |  |  |  |  |
| Waller et al. (2012) | ✓ |  |  | ✓ |  |  |  |  |
| Wang et al. (2016) | ✓ | ✓ | ✓ |  |  |  |  |  |
| Ward-Smith & Peterson (2016) | ✓ |  |  |  |  |  |  |  |
| Watson et al. (2008) |  |  |  |  |  |  |  | ✓ |
| Williams-Hailey (2015) | ✓ |  | ✓ |  |  |  |  | ✓ |
| Wise et al. (2014) |  |  |  | ✓ |  |  |  |  |
| Wynn et al. (2018) |  |  |  | ✓ |  |  |  |  |
| Yılmaz & Yabancı Ayhan (2018) | ✓ |  |  |  |  |  |  |  |
| Yılmaz & Yabancı Ayhan (2019) | ✓ |  | ✓ |  |  |  |  |  |
| Young (1985) | ✓ | ✓ | ✓ |  |  |  |  |  |
| Zhu et al. (2013) | ✓ |  |  |  |  |  | ✓ |  |
| Zuzelo & Seminara (2006) | ✓ | ✓ | ✓ |  |  |  |  |  |
